# Biomarkers of exercise-induced muscle damage and inflammation across menstrual cycle phases: a critical review

**DOI:** 10.3389/fspor.2026.1878668

**Published:** 2026-07-30

**Authors:** Farwa Baber, Jana Jaklová Dytrtová, Michal Jakl, Noor ul An Babar

**Affiliations:** Faculty of Physical Education and Sport, Sport Sciences–Biomedical Department, Charles University, Prague 6, Czechia

**Keywords:** eumenorrheic women, exercise-induced muscle damage, female athletes, inflammatory biomarkers, menstrual cycle, recovery

## Abstract

**Background:**

Menstrual cycle fluctuations in estradiol and progesterone may influence post-exercise biomarkers of muscle damage, inflammation, and recovery, but interpretation is limited by heterogeneous protocols, inconsistent phase verification, and poorly aligned sampling.

**Objective:**

To critically synthesize evidence on whether menstrual cycle phase modulates exercise-induced muscle damage, inflammatory, immune-cell, or recovery-related biomarker responses in naturally menstruating women not using hormonal contraception.

**Methods:**

PubMed, Scopus, and Web of Science were searched to June 21, 2026. Eligible studies assessed biomarkers or recovery outcomes after acute exercise or sport-related exposure across at least two menstrual cycle phases. Phase verification, exercise models, biomarker selection, sampling schedules, and methodological quality were synthesized narratively.

**Results:**

Eighteen studies were included. Exercise stimulus and sampling time were more consistent determinants of biomarker responses than menstrual phase labels alone. CK and IL-6 showed clear exercise-related, time-dependent responses, whereas phase-related differences were inconsistent across exercise models, endocrine contrasts, and verification methods. Emerging outcomes, including hepcidin, hs-CRP, oxidative-stress markers, immune-cell subsets, salivary IL-6, DOMS, and neuromuscular recovery, broadened the field but remained too heterogeneous for conclusions. Incomplete endocrine verification, non-equivalent phase definitions, small samples, and mismatched sampling limited interpretation.

**Conclusion:**

Current evidence does not support a uniform menstrual phase effect on biomarkers of exercise-induced muscle damage or inflammation. Menstrual status should be treated as a biologically relevant design and interpretation variable, not as a deterministic explanation for biomarker variation. Future studies require cycle-aware designs combining prospective tracking, ovulation confirmation, estradiol and progesterone assessment, standardized exercise stimuli, biomarker-specific sampling, and repeated-measures analyses.

## 1 Introduction

The menstrual cycle should be viewed not only as a reproductive rhythm but also as a dynamic endocrine context that may influence metabolism, stress responses, recovery, and exercise-related physiology. In sport and exercise research, increasing attention has therefore been directed toward how cyclical fluctuations in estradiol and progesterone may contribute to variability in exercise performance and biological responses to training and recovery in women (1–5). From an applied sport perspective, this interest has also been driven by the observation that many female athletes perceive fluctuations in performance across the cycle, even though objective performance outcomes remain inconsistent across studies (1). This issue is particularly relevant in female-specific sport science, where methodological shortcomings in menstrual cycle characterization have historically limited interpretation and comparability across studies.

In eumenorrheic women, the menstrual cycle is commonly divided into follicular and luteal phases, but this broad classification does not adequately capture the distinct endocrine milieus that occur across the cycle. More precise subphases, such as early follicular, late follicular or ovulatory, and mid-luteal, are often more informative because they differ substantially in estradiol and progesterone concentrations (2, 4, 5). This distinction is important because assigning participants to nominal menstrual phases based on cycle day alone may be misleading. Ovulation timing varies between individuals, phase terminology is not used consistently across studies, and calendar-based approaches or single-method phase estimation do not necessarily reflect the underlying hormonal state (2, 6). As a result, inadequate phase verification can obscure true hormone-related effects and reduce sensitivity to detect meaningful between-phase differences.

These limitations are especially important in sports research, where interest in menstrual cycle-based individualization has expanded more quickly than the quality of athlete-specific evidence. In elite female athletes, the available literature remains limited and heterogeneous, and current findings do not support clear evidence-based recommendations for performance optimization or training individualization based solely on menstrual cycle phase (7). At the same time, cycle-related variation may extend beyond strictly objective exercise measures, as some subjective and perceptual responses in athletes, including mood, fatigue, sleep quality, motivation, and competitiveness, appear to fluctuate across the cycle in at least some contexts (8). Together, these observations suggest that menstrual cycle-related variability may exist across multiple levels of response, but that its biological significance and practical relevance remain incompletely resolved.

Exercise-induced muscle damage and inflammation are two candidate domains in which menstrual cycle-related endocrine variation may be relevant. Exercise-induced muscle damage typically occurs after high-intensity, unaccustomed, or mechanically demanding exercise, particularly when the exercise includes a substantial eccentric component (9, 10). The initial mechanical disruption may be followed by secondary processes involving altered calcium handling, oxidative stress, and inflammatory signaling, which together shape post-exercise remodeling and recovery (10, 11). In human studies, these responses are commonly assessed using circulating biomarkers such as creatine kinase, myoglobin, lactate dehydrogenase, interleukin-6, and C-reactive protein (12). However, these biomarkers are indirect correlates rather than direct measures of local muscle damage, and their magnitude and time course are strongly influenced by exercise modality, intensity, training status, sampling design, and prior exposure to damaging exercise.

A mechanistic rationale for endocrine modulation of these responses does exist, but it is more complex than a simple “protective estrogen” model. Contemporary mechanistic work suggests that estradiol may influence exercise-induced muscle damage through both receptor-mediated signaling and structural antioxidant effects, including plasma membrane stabilization and attenuation of lipid peroxidation. Progesterone may further modify antioxidant defense, substrate use, and protein turnover, meaning that any apparent menstrual phase effect is likely to be context dependent and influenced by exercise modality, metabolic demands, and nutritional status (3, 13). At the same time, broader methodological heterogeneity across studies, especially differences in phase verification, exercise protocol design, and biomarker sampling schedules, continues to complicate interpretation (2, 6).

Consequently, it remains unclear whether reported between-phase differences in biomarkers of exercise-induced muscle damage and inflammation reflect true endocrine modulation or primarily methodological inconsistency. Clarifying this issue is important for both physiological interpretation and future study design. If ovarian hormone fluctuations meaningfully influence post-exercise biomarker responses, they may contribute to variability in findings across exercise studies in women. Conversely, if such effects are small or inconsistent relative to the influence of exercise characteristics and measurement design, this also has important implications for how future research should be conducted and interpreted. Therefore, this systematic review aimed to synthesize current evidence on whether menstrual cycle phase influences circulating biomarkers of exercise-induced muscle damage and inflammation in eumenorrheic women not using hormonal contraception. In addition to summarizing reported findings, the review evaluates key methodological features, particularly menstrual phase verification, exercise protocol characteristics, and biomarker sampling design, in order to identify priorities for more robust future research.

## 2 Methods

### 2.1 Study design and protocol

This systematic review was conducted and reported in accordance with the PRISMA 2020 statement. The review question was defined using a PICOS-informed framework: the population comprised eumenorrheic women of reproductive age not using hormonal contraception; the exposure was an acute exercise bout or sport-related exercise exposure expected to induce muscle damage and/or an inflammatory or immune response; the comparator was assessment across two or more menstrual cycle phases within the same study; the outcomes were biomarkers or immune-cell outcomes related to exercise-induced muscle damage, inflammation, or acute immune response measured at baseline and/or during post-exercise recovery; and eligible study designs included original studies using within-subject or phase-based between-group comparisons conducted within a single study. The full platform-specific database search strings, PICOS criteria, and modified Downs and Black appraisal items and scoring rules are provided in the Supplementary Information.

### 2.2 Search strategy and information sources

A systematic literature search was performed to identify studies examining whether menstrual cycle phase influences circulating biomarkers of exercise-induced muscle damage and/or inflammation following an acute exercise bout in eumenorrheic women not using hormonal contraception. PubMed, Scopus, and Web of Science Core Collection were searched from database inception to June 21, 2026. No additional non-database sources were used for study identification. Search terms combined concepts related to menstrual cycle phase, exercise, muscle damage, inflammation, and circulating biomarkers. No language restrictions were applied at the database-search stage. Document-type restrictions were applied in Web of Science and Scopus to retain original article records and early-access/article-in-press records where available; PubMed was searched without article-type restriction because publication-type indexing is not consistently equivalent to original research status. Eligibility criteria, including English full-text availability, original research design, naturally occurring menstrual cycles, and absence of hormonal contraceptive use, were applied during title/abstract and full-text screening. The complete platform-specific search strings are provided in the Supplementary Information.

### 2.3 Eligibility criteria

Studies were eligible for inclusion if they met all of the following criteria:

- were original peer-reviewed full-text articles published in English;
- included eumenorrheic women of reproductive age with regular natural menstrual cycles and no hormonal contraceptive use;
- involved an acute exercise bout, sport-related exercise exposure, or exercise protocol expected to elicit muscle damage and/or an inflammatory, immune, oxidative-stress, or recovery-related response;
- assessed at least one blood- or saliva-derived biomarker, circulating immune-cell outcome, or closely related recovery marker relevant to exercise-induced muscle damage, inflammation, or acute immune response at baseline and/or during post-exercise recovery;
- compared outcomes across two or more menstrual cycle phases within the same study, using either a within-subject design or a phase-based between-group comparison conducted within a single study; and
- reported extractable quantitative outcome data relevant to the review question.

Studies were excluded if they met any of the following criteria:

- included participants with menstrual cycle irregularities, menopause, pregnancy, or clinical conditions likely to influence endocrine function;
- included users of hormonal contraception without separately extractable data for naturally cycling non-users;
- were animal studies, *in vitro* studies, reviews, editorials, commentaries, or conference abstracts without accessible full text;
- lacked an accessible English full text; or
- did not report extractable numerical outcomes relevant to the review question.

### 2.4 Study selection

All records identified through the database searches were imported into Zotero (version 9) for reference management and deduplication. Duplicate records were identified using Zotero duplicate detection followed by iterative manual verification of bibliographic metadata. Because records from different databases were inconsistently indexed, manual verification considered title similarity, including differences caused by special characters, author-name formatting, publication year, journal, DOI, article number or other record identifiers, missing metadata, and early-access vs. final publication records. After duplicate removal, 944 unique records remained for title and abstract screening.

**Figure 1:**
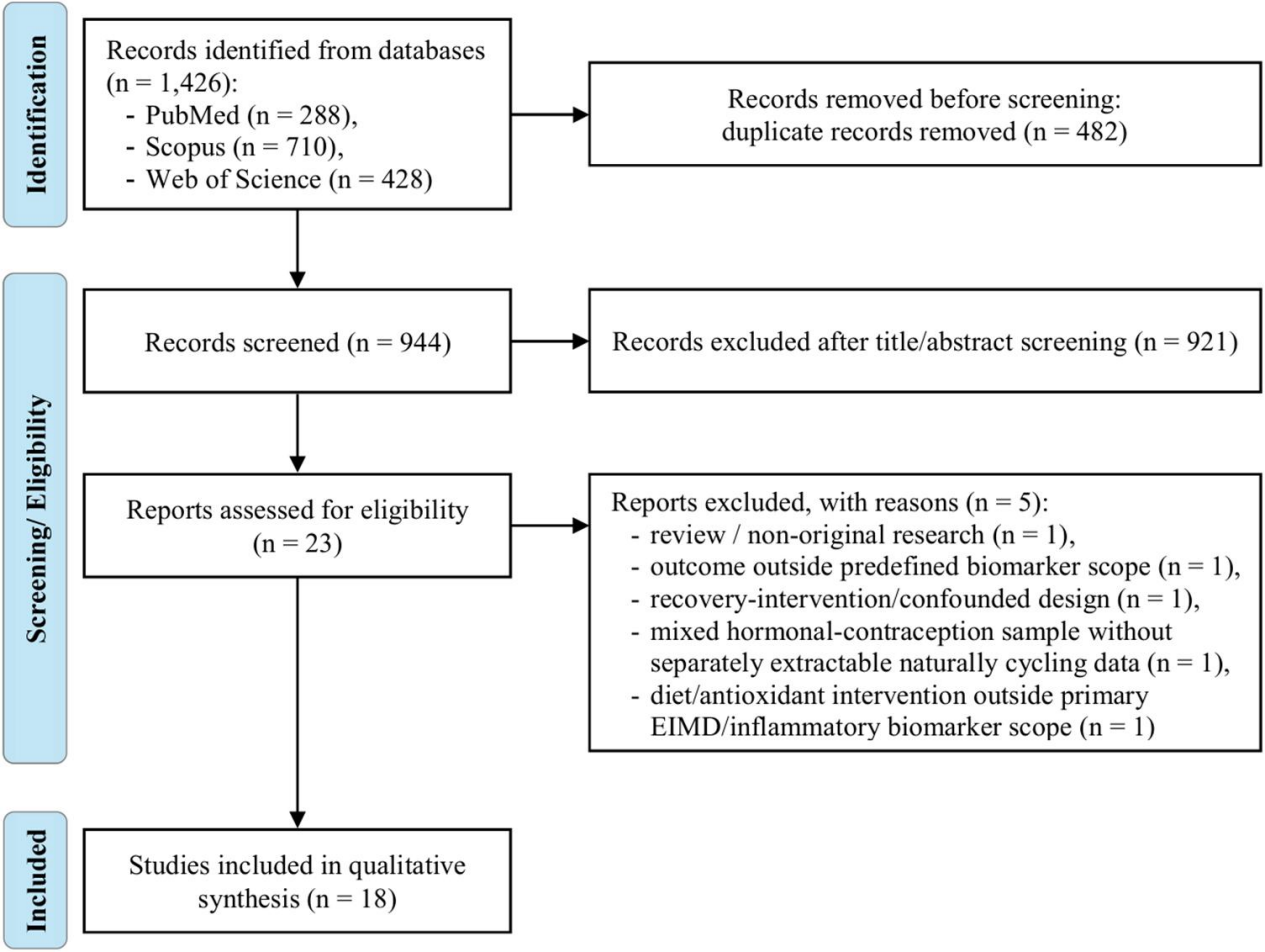
Flow diagram depicting phases of the search process and the subsequent study selection.

Titles and abstracts were screened against the predefined eligibility criteria. Records that clearly did not meet the review question were excluded at this stage, whereas records that appeared potentially eligible were retrieved and assessed in full text. Eligibility decisions were checked against the predefined PICOS criteria. Cases in which eligibility was uncertain were discussed among the review authors until consensus was reached. Formal inter-rater reliability statistics were not calculated because eligibility decisions and methodological quality ratings were resolved through consensus among the review authors rather than analyzed as independent reviewer classifications. The full study selection process, including the numbers of records identified, screened, excluded, assessed for eligibility, and included in the qualitative synthesis, is presented in the PRISMA flow diagram (Figure 1).

### 2.5 Data extraction

A standardized extraction form was used to ensure consistent and reproducible data collection across studies. The following data were extracted: study characteristics (authors, year, study design, sample size), participant characteristics (age, anthropometric variables, and training status or physical activity level), menstrual phase classification and verification procedures, exercise protocol characteristics (modality, intensity, duration, and/or volume), biomarker or recovery-related outcome type, biological matrix or sample type, reporting units or scale, post-exercise sampling schedule, and all extractable outcome values reported at baseline and recovery timepoints.

Because substantial heterogeneity existed in the timing of post-exercise measurements and in how outcomes were reported, sampling schedules and reporting formats were categorized descriptively to support the interpretation of between-study variability. For this purpose, post-exercise assessments were grouped as immediate protocols, short-term protocols, or extended recovery protocols, depending on the number and timing of sampling timepoints after exercise. Outcomes reported as model-based estimates, percentages, immune-cell subset changes, or recovery-related measures were retained in their original reporting format and synthesized narratively.

### 2.6 Methodological quality evaluation

Methodological quality was assessed using the modified Downs and Black checklist previously applied in menstrual cycle and exercise research (14). The full item-level checklist and scoring rules are provided in Supplementary Table S1A, and study-level scoring is provided in Supplementary Table S1B. Items Q1–Q15 were used to generate an *a priori* methodological quality score. The maximum possible score was 16 points, as Q9, relating to standardization of exercise testing conditions, was scored from 0 to 2, whereas the remaining scored items were rated as 0 or 1. *A priori* methodological quality was classified as high (13–16 points), moderate (9–12 points), low (5–8 points), or very low (0–4 points).

Because accurate menstrual phase classification was central to the review question, menstrual phase verification was incorporated as a predefined downgrade criterion rather than treated only as a descriptive limitation. Q16 and Q17 were not included in the 16-point *a priori* score. Instead, studies that did not confirm menstrual cycle phase using blood-based hormone assessment were downgraded by one level, and studies that did not confirm ovulation using prospective ovulation testing were downgraded by an additional level. Thus, the final methodological quality classification could be downgraded by up to two levels when menstrual phase verification was incomplete. Studies including hormonal contraceptive users without separately extractable data for naturally cycling non-users were considered ineligible rather than downgraded, because absence of hormonal contraceptive use was a predefined eligibility criterion.

In addition to the final methodological quality classification, a review-specific interpretive reliability summary was prepared for the qualitative synthesis. This summary considered whether biomarker sampling was aligned with expected biomarker kinetics, whether key confounders such as time of day, nutritional status, prior exercise, medication use, adiposity, and recovery behavior were controlled or reported, and whether the exercise protocol was appropriate for eliciting the target muscle damage or inflammatory response. These features are summarized in Table 1 and were used to guide the interpretive weight assigned to individual findings.

**Table 1:** Methodological quality and interpretive reliability of eligible studies reporting the modified downs and black score/final quality.

Study	Modified D&B score/final quality	Biomarker time-scale adequacy	Confounder control/reporting	Interpretive weight
Chaffin et al. (25)	11/16; Low (1 downgrade)	Partial: IL-6 only pre/∼5 min post; no delayed biomarker sampling.	Partial: training/body fat reported; nutrition, time of day, and prior exercise incompletely reported.	Low-moderate: useful for acute IL-6, but sparse sampling and small *n* limit inference.
Funaki et al. (23)	14/16; High	Adequate: pre, 4 h, 48 h, and 96 h captured CK and leukocyte kinetics.	Strong: fasting, alcohol/caffeine restriction, diet/sleep instructions, medication/supplement exclusions.	High: strong verification and time-scale alignment; between-group design limits within-subject inference.
Graja et al. (17)	15/16; High	Poor for CK: only pre and ∼3 min post; delayed CK kinetics not captured.	Strong: morning testing, overnight fast, standardized breakfast, familiarization, exercise restriction, lifestyle/diet instructions.	Moderate: strong phase verification, but immediate-only CK sampling limits muscle-damage interpretation.
Hackney et al. (26)	11/16; Low (1 downgrade; HC exclusion unclear)	Adequate: immediate, 24 h, and 72 h captured extended CK/IL-6 recovery.	Moderate: diet and prior exercise controlled; short report gives limited detail; small pilot sample.	Moderate-cautious: good recovery sampling, but small n, no ovulation testing, and unclear HC exclusion reduce certainty.
Minuzzi et al. (31)	14/16; Low (2 downgrades)	Adequate for acute cytokines: pre, post, and 1 h post.	Moderate-strong: prior exercise, ergogenic/alcohol restriction, and standardized breakfast reported.	Low-moderate: useful cytokine kinetics, but weak phase verification limits menstrual-phase inference.
Romero-Parra et al. (21)	14/16; High	Good: pre, 2 h, 24 h, and 48 h covered multiple markers; very late CK peak may still be missed.	Strong: DEXA body composition, randomized/counterbalanced sessions, power calculation, standardized eccentric protocol.	High: strongest evidence for eccentric resistance exercise with robust verification and multi-marker sampling.
Sawai et al. (22)	12/16; Moderate	Adequate for acute IL-6/cfDNA: pre, immediate, 30 min, and 60 min post.	Strong: morning fasted testing, alcohol/caffeine restriction, body composition, VO_2_max; final sample small.	Moderate: strong phase verification and acute sampling, but the small final sample and participant loss during hormonal verification limit certainty; moderate cycling is not a classic EIMD stimulus.
Sipavičienė et al. (29)	12/16; Low (1 downgrade)	Adequate for CK/function: delayed measures up to 72 h.	Partial: randomized order and >=10-week interval; nutrition, time of day, medication, and adiposity incompletely reported.	Low-moderate: useful delayed CK sampling, but incomplete endocrine verification lowers certainty.
Williams et al. (30)	12/16; Low (1 downgrade)	Partially adequate for CK: pre, immediate, 30 min, and 24 h; later CK peak may be missed.	Moderate: food diary/similar diet, prior exercise restriction, anti-inflammatory medication exclusion, body fat, plasma volume correction.	Moderate: relevant CK outcome, but E2-only verification and no ovulation confirmation limit phase attribution.
Parsons et al. (20)	11/16; Low (1 downgrade)	Adequate for hs-CRP: GD-1, GD + 1, GD + 2, and GD + 3 align with delayed CRP recovery.	Strong for field context: fasted morning sampling, BMI, fatigue, minutes played, MC symptoms, sport type, injury/illness, NSAIDs considered.	Moderate: strong recovery sampling and covariate modelling; no blood hormones and field variability limit causal inference.
Oosthuyse & Bosch (19)	14/16; High	Adequate for CK: pre, immediate, 24 h, 48 h and 72 h captured the delayed CK response after downhill running.	Strong: oral temperature/ovulation kit and serum hormones; exercise time controlled; prior strenuous exercise, alcohol, antioxidant supplements and medication restricted.	Moderate-high: strong phase verification and appropriate CK sampling; between-group phase design and small *n* per phase limit inference.
Barba-Moreno et al. (16)	13/16; Moderate (1 downgrade)	Adequate for acute IL-6 and hepcidin: pre, 0 h and 3 h post-exercise; not designed for delayed CK-type kinetics.	Moderate-strong: fasted baseline screening, DEXA and VO_2_peak; hormone measures supported phase assignment; no LH-surge testing.	Moderate: relevant IL-6/hepcidin design and useful early sampling; no ovulation testing and iron-focused scope limit direct EIMD interpretation.
Alfaro-Magallanes et al. (15)	14/16; High	Good for IL-6/hepcidin and iron regulation: pre, 0 h, 3 h and 24 h post-exercise.	Strong: three-step menstrual verification, blood hormones, power calculation, standardized interval running and repeated-measures design.	High for inflammatory/iron-regulatory response; hepcidin/iron focus should be interpreted as adjacent to inflammation rather than direct muscle damage.
Zheng et al. (24)	14/16; High	Adequate for IL-6/hepcidin: pre, post and 3 h post-exercise; not designed for delayed muscle-damage markers.	Strong: urinary LH plus serum E2/P4, dietary/exercise control, morning trials, randomized/counterbalanced environmental conditions; final *n* small after exclusions.	Moderate-high: robust verification and controlled crossover; heat-stress/performance context and small final *n* limit generalizability to EIMD.
Gassner et al. (28)	14/16; Low (2 downgrades)	Adequate for acute IL-6/ROS only: baseline, pre-HIIT, post-HIIT and 15 min post-HIIT; no delayed recovery sampling.	Moderate: fasted standardized testing and 48 h activity/alcohol restriction; menstrual phase classified mainly by calendar/self-report, with estradiol but no progesterone or ovulation confirmation.	Low-moderate: useful acute HIIT IL-6/ROS data, but weak phase verification and small subgroup sizes strongly limit phase attribution.
Hicks et al. (18)	14/16; Moderate (1 downgrade)	Adequate for immediate immune-cell mobilization; no delayed inflammatory recovery sampling.	Moderate: prior exercise, diet timing and stimulants controlled; serum E2/P4 measured; no ovulation testing; very small sample.	Moderate: useful exploratory immune-cell evidence across three phases; preprint/brief-report format and small *n* require caution.
Silva et al. (32)	14/16; Low (2 downgrades)	Partially adequate for recovery: rest, post, 24 h and 48 h captured IL-6/DOMS/RSI after SSGs; salivary IL-6 and no blood biomarkers.	Partial: regular diet/training maintained and sessions structured, but sleep/nutrition/stress not tightly controlled; phase based on app/calendar only.	Low-moderate: applied sport-recovery data are relevant, but calendar-only phase classification and salivary IL-6 reduce causal interpretation.
Yoshida et al. (27)	13/16; Moderate (1 downgrade)	Adequate for acute IL-6/d-ROMs: pre, immediate post and 60 min post-exercise; not designed for delayed recovery markers.	Moderate: fasted trials, time window and prior exercise/alcohol restrictions; E2/P4 measured; no ovulation testing and MC phase was secondary to sex-difference/workload question.	Moderate-low: informative for workload-adjusted IL-6 interpretation, but menstrual phase was not the primary analytical focus.

Modified D&B score refers to Q1–Q15 of the modified Downs and Black checklist in Supplementary Tables S1A and S1B. HC, hormonal contraception; CK, creatine kinase; CRP, C-reactive protein; GD, game day; IL-6, interleukin-6; cfDNA, cell-free DNA; EIMD, exercise-induced muscle damage.

### 2.7 Data synthesis

A qualitative (narrative) synthesis was conducted because the included studies were substantially heterogeneous in participant training status, menstrual phase definitions and verification procedures, exercise modality and intensity, biomarker or recovery-related outcome selection, biological matrix, and post-exercise sampling schedules. This degree of methodological and clinical heterogeneity precluded meaningful quantitative pooling. No targeted quantitative sub-analysis was performed because no subset combined sufficiently comparable menstrual phase contrasts, exercise stimuli, biomarker domains, biological matrices, and post-exercise sampling timepoints to support meaningful pooling without introducing misleading assumptions.

The synthesis focused on whether evidence for menstrual cycle phase–related differences was consistent across studies and interpreted findings in relation to the methodological factors most likely to affect comparability. In particular, emphasis was placed on three domains: (i) menstrual phase verification, (ii) exercise protocol characteristics, and (iii) biomarker/outcome sampling design. Greater interpretive weight was given to studies with stronger methodological quality, especially those using biochemical confirmation of menstrual phase, prospective ovulation testing, and more appropriate temporal alignment between outcome measurement and expected post-exercise response.

## 3 Results

### 3.1 Study selection

The database searches identified 1,426 records: 288 from PubMed, 710 from Scopus, and 428 from Web of Science Core Collection. After the removal of 482 duplicate records, 944 records remained for title and abstract screening. Of these, 921 records were excluded after title and abstract screening, and 23 full-text reports were assessed for eligibility. Five full-text reports were excluded: one review or non-original research article, one report with outcomes outside the predefined biomarker scope, one recovery-intervention or confounded design, one mixed hormonal-contraception sample without separately extractable naturally cycling data, and one diet or antioxidant intervention outside the primary exercise-induced muscle damage/inflammatory biomarker scope. Eighteen studies met the eligibility criteria and were included in the qualitative synthesis. The full study selection process is shown in Figure 1.

### 3.2 Study characteristics and menstrual-phase verification

The characteristics of the included studies are summarized in Table 2. The 18 included studies enrolled between 7 and 30 women. Participant populations included sedentary or untrained women, physically active or recreationally active women, endurance-trained women, runners, handball players, soccer players, and recreational female athletes participating in field-based sport exposure.

**Table 2:** Characteristics of the sample, training status, and determination of cycle phase.

Authors	Sample size	Age (years)	Height (cm)	Weight (kg)	Body Fat (%)	BMI (kg/m^2^)	VO_2_max (mL/kg/min)	Training status	Determination of cycle phase
Sawai et al. (22)	7	24.8 ± 1.5	159.0 ± 1.1	52.8 ± 2.0	—	—	42.1 ± 3.9	Sedentary females	Ovulation testing kit, E2 and P4 measurements
Funaki et al. (23)	24	26.5 ± 6.5	159.5 ± 5	56.5 ± 7.3	—	22.2 ± 2.4	—	Untrained females	Ovulation testing kit, E2 and P4 measurements
Minuzzi et al. (31)	14	24 ± 2	—	—	—	22.8 ± 1.9	—	Physically active females	Self-reported
Romero-Parra et al. (21)	19	28.6 ± 5.9	163.4 ± 6.1	59.6 ± 5.8	14.8 ± 5.1	22.8 ± 1.9	—	Well-trained females	Ovulation testing kit, E2 and P4 measurements
Graja et al. (17)	10	22.5 ± 1.5	170 ± 4	LFP: 65.2 ± 8.0	—	—	—	Well-trained handball females	Menstruation diary record for minimum 6 months, ovulation test strip. Serum estrogen and progesterone were used to verify physiological reproductive function and verify timing of tests with respect to the MP
				MLP: 65.5 ± 7.3					
				PMP: 66.1 ± 6.4					
Hackney et al. (26)	8	25 ± 4	167.8 ± 3.1	58.9 ± 3.7	22.5 ± 4.4	—	57.5 ± 3.5	Trained female runner	E2 & P4 measurements
Williams et al. (30)	10	21 ± 1	164.5 ± 5.7	61.3 ± 8.3	24.0 ± 3.0	—	53.5 ± 4.7	Highly trained females	E2 measurements
Sipavičienė et al. (29)	18	20.2 ± 1.7	167.3 ± 3.2	56.2 ± 4.1	—	—	—	Physically active females	E2 measurement, rectal temperature recorded (to access menstrual cycle phases) for three months
Chaffin et al. (25)	9	26.8 ± 4.3	166.1 ± 6.1	57.2 ± 4.2	19.1 ± 4.4	—	49.7 ± 6.0	Competitive physical active females	E2 & P4 measurements, documentation of previous cycles and temperature recordings to determine their cycle length
Parsons et al. (20)	19	21.3 ± 2.9	—	—	—	22.1 ± 1.5	—	Recreational female athletes; at least trained/developmental level; weekly team-sport matches or individual sports fixtures	Self-reported bleed data combined with urinary ovulation testing, No blood-based E2/P4 confirmation
Oosthuyse & Bosch (19)	15	21.7 ± 2.4	160.6 ± 5.5	58.0 ± 10.2	—	22.4 ± 3.4	—	Sedentary females	Oral temperature for one cycle, home ovulation kit, and serum E2/P4 confirmation
Barba-Moreno et al. (16)	15	25—40 (mean not reported)	—	—	—	—	—	Healthy endurance-trained/active females (5–12 h endurance sports/week)	Cycle history/calendar calculation by gynecologist plus venous E2, P4, LH and FSH; no LH-surge testing
Alfaro-Magallanes et al. (15)	21	30.5 ± 6.5	163.0 ± 6.0	58.4 ± 8.7	26.4 ± 7.0	—	48.4 ± 4.4	Endurance-trained females; trained/developmental level	Calendar-based scheduling by gynecologist, urinary LH testing, and blood E2/P4/LH/FSH/prolactin confirmation
Zheng et al. (24)	8	37 ± 7	166 ± 9	63 ± 7	22 ± 6	—	46 ± 7	Trained, iron-sufficient females	Three-step approach: self-reported menses onset, urinary LH testing, and retrospective serum E2/P4 confirmation; anovulatory cycles excluded
Gassner et al. (28)	30	18—40 eligible (mean not reported)	—	—	—	18.5—25 eligible	—	Young healthy sedentary females; <2 h physical activity/week; no regular resistance training	Prospective self-tracking/calendar-based phase classification with serum estradiol measured; no progesterone or ovulation confirmation
Hicks et al. (18)	7	27 ± 3	170 ± 6	68 ± 6	—	—	—	Recreationally active	Calendar counting for D4/D14/D21 with retrospective serum estrogen and progesterone measurement; no ovulation testing
Silva et al. (32)	20	21.4 ± 1.8	158.2 ± 4.7	53.5 ± 3.1	—	20.8 ± 0.9	—	Amateur female soccer players; mean 3.4 ± 0.9 years training experience	Self-reported menstrual tracking using Clue app; early follicular and mid-luteal phase based on calendar estimates; no biochemical confirmation
Yoshida et al. (27)	13	25.7 ± 2.8	160.3 ± 5.9	54.5 ± 3.0	—	21.5 ± 2.0	—	Healthy non-athlete females	Self-reported recent cycle records; MP days 1–5 and LP latter half of cycle; serum E2/P4 measured; no ovulation testing

The included studies compared two or more menstrual cycle phases within the same study. Eight studies included three or more phase categories: Alfaro-Magallanes et al. (15), Barba-Moreno et al. (16), Graja et al. (17), Hicks et al. (18), Oosthuyse & Bosch (19), Parsons et al. (20), Romero-Parra et al. (21), Sawai et al. (22). The remaining studies compared two phase conditions.

Menstrual-phase verification methods differed across studies. Seven studies used both blood-based ovarian hormone assessment and prospective ovulation testing: Alfaro-Magallanes et al. (15), Funaki et al. (23), Graja et al. (17), Oosthuyse & Bosch (19), Romero-Parra et al. (21), Sawai et al. (22), and Zheng et al. (24). Five studies measured blood-based ovarian hormones without prospective ovulation testing: Barba-Moreno et al. (16), Chaffin et al. (25), Hackney et al. (26), Hicks et al. (18), and Yoshida et al. (27). Three studies used partial blood-based hormone assessment without both estradiol and progesterone confirmation or without ovulation testing: Gassner et al. (28), Sipavičienė et al. (29), and Williams et al. (30). Parsons et al. (20) used self-reported bleeding data together with urinary ovulation testing but did not include blood-based estradiol or progesterone confirmation. Minuzzi et al. (31) and Silva et al. (32) relied on self-reported or app/calendar-based menstrual-cycle timing without biochemical hormone confirmation or ovulation testing.

### 3.3 Exercise protocols, outcomes, and biomarker findings

The exercise protocols, measured outcomes, sampling schedules, and main findings are summarized in Table 3. Exercise models included eccentric resistance exercise, stretch-shortening exercise, downhill running, prolonged treadmill running, interval or sprint running, cycling protocols, resistance circuit HIIT, cycling time trials, soccer small-sided games, and field-based game-day sport exposure.

**Table 3:** Overview of study variables and outcomes.

Author	Compared menstrual cycle phases/day	Hormone concentrations E2 (pg/mL)	Hormone concentrations P4 (ng/mL)	Biomarkers/recovery-related outcomes and sampling schedule (selected units: CK, LDH: U/L; CK-MB: ng/mL; IL-6, TNF-α: pg/mL)	Exercise	Results
Sawai et al. (22)	Menstruation (day 1–4)	pre; ∼30	pre; ∼0.3	IL-6 pre; 1.1 (0.3)	10 min warmup at an intensity of 40 W & 60 rpm, test performed a pedaling exercise for 30 min at 60% VO_2_max intensity	IL-6 increased after moderate-intensity cycling, peaked at 30 min post-exercise, and declined by 60 min. The main effect of time was significant, whereas the main effect of phase was not significant and the phase × time interaction did not reach statistical significance.
		post 0; ∼45	post 0; ∼0.3	IL-6 post 0; 1.4 (0.3)		
		post 30 min; ∼45	post 30; min ∼0.3	IL-6 post 30 min; 2.0 (0.4)		
		post 60 min; ∼40	post 60 min; ∼0.3	IL-6 post 60 min; 1.3 (0.3)		
	OP (within 2 days, the day of ovulatory detected)	pre ∼120	pre; ∼1.5	IL-6 pre; 1.2 (0.3)		
		post 0 ∼150	post 0; ∼1.5	IL-6 post 0; 1.5 (0.4)		
		post 30 min ∼140	post 30 min; ∼1.5	IL-6 post 30 min; 2.7 (0.5)		
		post 60 min: ∼120	post 60 min; ∼1.5	IL-6 post 60 min; 1.6 (0.4)		
	LP (5–10 days before next expected menstrual onset date)	pre; ∼170	pre; ∼18	IL-6 pre; 0.9 (0.3)		
		post 0; ∼210	post 0; ∼22	IL-6 post 0; 1.2 (0.4)		
		post 30 min; ∼180	post 30 min; ∼19	IL-6 post 30 min; 1.6 (0.4)		
		post 60 min: ∼150	post 60 min ∼18	IL-6 post 60 min; 1.2 (0.3)		
Funaki et al. (23)	EFP (4.7 ± 2.0 days)	37.5 [30.8–50.8]	0.2 [0.2–0.3]	CK pre; 79.5 (54.5–89.3)	isokinetic dynamometer—exercise comprised 10 sets of six maximal voluntary isokinetic eccentric exercises of elbow flexors at a velocity of 90/s for ROM from 90 to 0 flexion, indicating a full extension	CK increased markedly after eccentric elbow-flexor exercise and peaked at 96 h in both EFP and MLP groups. The magnitude of CK increase did not differ significantly between phases.
				CK 4 h; 82.0 (62.0–90.3)		
				CK 48 h;314.0 (102.5–649.3)		
				CK 96 h; 3,997.5 (228.6–7,712.3)		
	MLP (24.2 ± 3.4 days)	171.0 [120.5–245.3]	14.4 [12.4–17.4]	CK pre; 64.5 (56.0–81.3)		
				CK 4 h; 76.5 (58.5–87.8)		
				CK 48 h; 199.0 (78.3–1,496.0)		
				CK 96 h; 5,893.5 (216.3–10,195.3)		
Minuzzi et al. (31)	Follicle (∼day 7)	—	—	IL-6 pre; 2.4 ± 0.25	4 sessions: 2 during FP and 2 during LP. 1st exercise session—to determine VO_2_max and MAV, a max incremental test (initial speed of 6.5 km/h with an increase of 1.5 km/h every 2 min until volunteer exhaustion)	IL-6 and TNF-α changed over time after high-intensity intermittent exercise, but no phase × time interaction was observed. Resting TNF-α was higher in the luteal phase, whereas exercise-induced cytokine changes were primarily time-dependent.
				IL-6 IP; 2.2 ± 0.2		
				IL-6 1 h; 1.2 ± 0.2		
				TNF-α pre; 5.0		
				TNF-α post; 4.5		
				TNF-α 1 h; 2.5		
	Luteal (∼day 21)	—	—	IL-6 pre; 2.7 ± 0.25		
				IL-6 IP; 2.0 ± 0.25		
				IL-6 60 min; 1.1 ± 0.1		
				TNF-α pre; 10		
				TNF-α post; 7.8		
				TNF-α 1 h; 2.5		
Romero-Parra et al. (21)	EFP (3 ± 1 days)	38.2 ± 32.1	0.3 ± 0.1	CK pre; 108.6 ± 48.0	5-min cycle-ergometer warm-up and some mobility and dynamic stretching exercises, the 1RM for the back-squat exercise, eccentric-method: 10 sets of 10 reps of plate-loaded barbell parallel back squats, at 60% of their 1RM, with 2 min of rest between sets	CK, myoglobin, and LDH changed over time after eccentric squat exercise but did not differ significantly across EFP, LFP, and MLP. IL-6 showed a time × phase interaction, with higher 2 h post-exercise values in MLP than in the follicular phases.
				CK post 2 h; 151.6 ± 70.0		
				CK post 24 h; 154.1 ± 69.3		
				CK post 48 h; 117.3 ± 40.1		
				IL-6 pre; 1.7 ± 0.7		
				IL-6 post 2 h; 1.8 ± 0.7		
				IL-6 post 24 h; 1.6 ± 0.2		
				IL-6 post 48 h; 1.6 ± 0.5		
				LDH pre; 166.5 ± 14.7		
				LDH post 2 h; 187.1 ± 20.3		
				LDH post 24 h; 166.2 ± 15.9		
				LDH post 48 h; 163.8 ± 16.5		
	LFP (12 ± 3 days)	185.1 ± 173.9	0.4 ± 0.7	CK pre; 105.7 ± 33.1		
				CK post 2 h; 155.1 ± 44.9		
				CK post 24 h; 195.5 ± 95.3		
				CK post 48 h; 130.6 ± 47.7		
				IL-6 pre; 1.7 ± 0.5		
				IL-6 post 2 h; 1.7 ± 0.6		
				IL-6 post 24 h; 1.7 ± 0.7		
				IL-6 post 48 h; 1.9 ± 0.7		
				LDH pre; 164.3 ± 22.6		
				LDH post 2 h; 181.5 ± 26.8		
				LDH post 24 h; 170.2 ± 23.9		
				LDH post 48 h; 161.4 ± 16.2		
	MLP (22 ± 3 days)	156.1 ± 91.5	10.1 ± 3.9	CK pre; 100.7 ± 29.9		
				CK post 2 h; 150.1 ± 43.8		
				CK post 24 h; 172.1 ± 85.8		
				CK post 48 h; 128.8 ± 49.5		
				IL-6 pre; 1.6 ± 0.3		
				IL-6 post 2 h; 2.0 ± 1.3		
				IL-6 post 24 h; 1.5 ± 0.0		
				IL-6 post 48 h; 1.5 ± 0.1		
				LDH pre; 163.9 ± 13.4		
				LDH post 2 h; 184.4 ± 16.3		
				LDH post 24 h; 165.7 ± 17.4		
				LDH post 48 h; 167.9 ± 16.9		
Graja et al. (17)	LFP (11–13 days)	386.9 ± 31.9	0.54 ± 0.18	CK pre; 95.8 ± 10.1	20 × 5-s cycle sprints interspersed with 25 s of rest	CK increased immediately after repeated sprint exercise in all phases and was higher in PMP than in LFP or MLP. Because CK was assessed only pre- and immediately post-exercise, delayed CK kinetics were not captured.
				CK post; 113.6 ± 12.4		
	MLP (21–23 days)	194.0 ± 6.0	14.61 ± 1.68	CK pre; 98.8 ± 8.8		
				CK post; 119.1 ± 7.8		
	PMP (28–29 days)	77.5 ± 6.5	1.77 ± 0.62	CK pre; 99.5 ± 9.2		
				CK post; 127.2 ± 11.5		
Hackney et al. (26)	MFP (8 ± 2 days)	23.8 ± 6.6	1.1 ± 0.4	CK pre; 89.7 ± 16.7	Running for 90 min at 70% maximal oxygen uptake (VO_2_max) on a treadmill. Running speed was 13.0 ± 0.5 km/h	CK and IL-6 increased after prolonged treadmill running and showed greater recovery responses in MFP than in MLP, particularly at 24 h and 72 h. These findings suggest a phase-related difference, but interpretation is limited by the small pilot sample and absence of ovulation testing.
				CK IP; 109.7 ± 10.7		
				CK 24 h; 510.8 ± 344.9		
				CK 72 h; 425.7 ± 249.7		
				IL-6 pre; 1.4 ± 1.9		
				IL-6 IP; 24.9 ± 13.2		
				IL-6 24 h; 10.3 ± 7.1		
				IL-6 72 h; 4.3 ± 3.1		
	MLP (23 ± 3 days)	134.3 ± 38.8	6.7 ± 3.9	CK pre; 92.6 ± 9.6		
				CK IP; 112.7 ± 16.8		
				CK 24 h; 275.1 ± 55.1		
				CK 72 h; 425.7 ± 249.7		
				IL-6 pre; 1.2 ± 0.5		
				IL-6 IP; 13.5 ± 6.2		
				IL-6 24 h; 5.0 ± 3.0		
				IL-6 72 h; 0.9 ± 0.3		
Williams et al. (30)	MFP (7 ± 2 days)	39.8 ± 18.3	—	CK pre; 106.8 ± 34.5	VO_2_max continuous, mental treadmill protocol on a Quinton Q65 treadmill. 5-min warm up, resting VO_2_ was measured for 3 min, next the exercise test using the Bruce treadmill protocol	Total CK increased after treadmill exercise and was higher in MFP than in MLP, particularly at 24 h post-exercise. CK-MB did not differ by hormonal condition or time, suggesting that the phase effect did not generalize across related enzyme markers.
				CK IP; 129.9 ± 38.0		
				CK 30 min; 161.2 ± 60.9		
				CK 24 h; 378.7 ± 176.5		
				CK-MB pre; 4.7 ± 3.4		
				CK-MB IP; 4.8 ± 1.4		
				CK-MB 30 min; 6.2 ± 1.5		
				CK-MB 24 h; 5.6 ± 2.0		
	MLP (23 ± 3 days)	148.1 ± 35.2	—	CK pre; 104.2 ± 3.1		
				CK IP; 118.6 ± 18.7		
				CK 30 min; 153.4 ± 28.9		
				CK 24 h; 279.6 ± 100.8		
				CK-MB pre; 5.0 ± 2.3		
				CK-MB IP; 6.0 ± 2.3		
				CK-MB 30 min; 5.4 ± 1.0		
				CK-MB 24 h; 5.7 ± 1.4		
Sipavičienė et al. (29)	EFP (1–2 days)	32.4 ± 16.8	—	CK pre; 1 ± 0.2	100 maximum drop jumps from a height of 0.75 m from a platform	Stretch-shortening exercise increased CK in both EFP and late follicular/ovulatory testing. No significant between-phase difference in CK response was observed.
				CK 24 h; 12 ± 6		
				CK 48 h; 7 ± 4		
				CK 72 h; 5 ± 2		
	LFP (1–2 days of ovulatory phase)	62.5 ± 22.3	—	CK pre; 1 ± 0.2		
				CK 24 h; 9 ± 3		
				CK 48 h; 5 ± 2		
				CK 72 h; 4 ± 1.5		
Chaffin et al. (25)	EFP (1–3 days)	68.4 ± 28.7 [18.98–98.28]	8.7 ± 3.4 [2.04–13.03]	IL-6 pre; 0.8 ± 0.8	Two rounds of: 3 × (2 min running at 100% V˙O_2_ peak with 2 min jogging or walking at 50% V˙O_2_ peak) with 3 min walk between rounds; 5 min recovery at a self-selected pace; 5× (1 min running at 110% V˙O_2_ peak + 3 min walk)	IL-6 increased from pre- to post-exercise after interval running. No significant menstrual phase effect and no phase × time interaction were observed.
				IL-6 post; 4.6 ± 2.5		
	MLP (20–22 days)	85.8 ± 14.8 [73.95–106.68]	13.7 ± 2.5 [9.00–16.63]	IL-6 pre; 0.8 ± 1.0		
				IL-6 post; 6.1 ± 3.5		
Parsons et al. (20)	EFP (cycle day not reported)	Not measured	Not measured	hs-CRP assessed at GD−1, GD + 1, GD + 2, and GD + 3. Phase-specific model estimate showed hs-CRP 24.20% higher on GD + 1 compared with GD−1	Real-world weekly game-day exposure across recreational female athletes from team and individual sports	hs-CRP showed a game day × menstrual phase interaction, with the largest GD + 1 increase estimated in LLP. No significant phase-specific difference was reported for GD + 2 or GD + 3.
	LFP (cycle day not reported)	Not measured	Not measured	hs-CRP assessed at GD−1, GD + 1, GD + 2, and GD + 3. Phase-specific model estimate showed hs-CRP 15.93% higher on GD + 1 compared with GD−1		
	MLP (cycle day not reported)	Not measured	Not measured	hs-CRP assessed at GD−1, GD + 1, GD + 2, and GD + 3. Phase-specific model estimate showed no notable GD + 1 increase in hs-CRP compared with GD−1		
	LLP (cycle day not reported)			hs-CRP assessed at GD−1, GD + 1, GD + 2, and GD + 3. Phase-specific model estimate showed hs-CRP 64.65% higher on GD + 1 compared with GD−1		
Oosthuyse & Bosch (19)	EFP (day 3.6 ± 1.1)	45.2 ± 24.2	0.72 ± 0.28	Serum CK assessed pre-exercise, immediately post, 24 h, 48 h and 72 h; perceived muscle soreness also assessed	20 min downhill treadmill running at 9 km/h and -10% gradient	CK peaked at 24 h after downhill running in EFP, LFP, and MLP groups and returned toward baseline by 48 h. Pre-exercise CK did not differ by phase, and the CK response was not consistently associated with serum estrogen when all participants were included.
	LFP (two days before ovulation to ovulation; day 14.4 ± 2.7)	269.9 ± 110.8	1.29 ± 0.47			
	MLP (4–8 days after ovulation; day 20.4 ± 2.2	136.2 ± 67.2	8.15 ± 2.58			
Barba-Moreno et al. (16)	EFP (day 3 ± 0.9)	39.4 ± 18.4	0.91 ± 0.79	IL-6 and hepcidin assessed pre, 0 h and 3 h post-exercise. Iron, ferritin, transferrin and CRP were also assessed	40 min treadmill running at 75% vVO_2_peak	IL-6 showed a menstrual phase × time interaction, with higher 3 h post-exercise values in LP than in EFP or MFP. Hepcidin did not differ significantly across menstrual phases.
	MFP (day 8 ± 1.1)	82.7 ± 51.3	0.53 ± 0.31			
	LP (day 21 ± 1.9)	110.7 ± 33.6	10.43 ± 5.58			
Alfaro-Magallanes et al. (15)	EFP (3.4 ± 0.9 days)	38.8 ± 30.4	0.33 ± 0.19	Hepcidin-25, IL-6, TNF-α, CRP, iron, ferritin, transferrin and TSAT assessed pre-exercise, 0 h, 3 h and 24 h post-exercise	8 × 3 min running bouts at 85% maximal aerobic speed, with 90 s recovery at 30%; 5 min warm-up and 5 min cool-down.	Hepcidin showed a time effect but no significant menstrual phase effect or phase × time interaction. IL-6 and TNF-α increased over time without a clear phase effect, while iron-related markers differed across phases, with lower iron availability in EFP.
	LFP (12.0 ± 2.5 days)	186.7 ± 156.3	0.75 ± 1.79			
	MLP (21.9 ± 3.1 days)	138.1 ± 72.0	12.00 ± 5.37			
Zheng et al. (24)	EFP (testing days 3 ± 1 and 7 ± 2)	79 ± 62	0.6 ± 0.3	IL-6 and hepcidin assessed pre-exercise, post-exercise and 3 h post-exercise; leukocytes, platelets, ferritin, transferrin, iron, TSAT and iron binding also assessed	20 min fixed-intensity cycling (100 W and 125 W) followed by 30 min self-paced cycling work trial at ∼75% VO_2_max in moderate and warm-humid environments	IL-6 and hepcidin increased after cycling, but neither response was affected by menstrual phase or ambient condition. IL-6 was mainly related to leukocyte and platelet responses, whereas hepcidin was related to iron and ferritin.
	MLP (testing days 20 ± 2 and 22 ± 3)	116 ± 85	13.8 ± 6.4			
Gassner et al. (28)	FP (calendar/self-tracked classification)	phase-specific E2 not reported for FP/LP in main table	not measured	ROS and plasma IL-6 assessed at baseline, pre-HIIT, post-HIIT and 15 min post-HIIT. FP IL-6: baseline 1.55 ± 1.05; pre 1.20 ± 0.85; post 1.80 ± 1.11; 15-post 1.37 ± 0.70	Resistance circuit HIIT: rowing, chest press, leg curl, lat pulldown and leg press; 40 s work/10 s rest; three rounds; 50% estimated 1RM; fasted state	IL-6 and ROS showed time-dependent responses around resistance circuit HIIT. Responses did not differ by menstrual phase, estradiol group, or menstruation status.
	LP (calendar/self-tracked classification	phase-specific E2 not reported for FP/LP in main table	not measured	LP IL-6: baseline 1.16 ± 0.81; pre 1.15 ± 0.67; post 1.89 ± 1.29; 15-post 1.54 ± 0.80. ROS also assessed at the same timepoints		
Hicks et al. (18)	EFP (day 4 ± 1)	day 14 higher than day 4	day 21 higher than day 4	Peripheral blood mononuclear cell subsets assessed pre and immediately post 5 km cycling time trial using flow cytometry, including naive and effector-memory T-cells, Tregs, NK cells and monocytes expressing TLR4	5 km cycling time trial on a cycle ergometer following a 2 min warm-up at 80 W	Exercise-induced reductions in selected PBMC subsets were most evident in EFP and were attenuated or absent in LFP/OP and MLP. These findings suggest phase-related variation in acute immune-cell redistribution.
	LFP/OP (day 14 ± 1)		low relative to day 21			
	MLP (day 21 ± 1)	Measured	day 21 higher than day 4			
Silva et al. (32)	EFP (days 2–5)	Not measured	not measured	Salivary IL-6, DOMS and reactive strength index assessed at rest, immediately post-session, 24 h and 48 h after 1v1 and 5v5 small-sided games. EFP IL-6: 1v1 rest 0.47 ± 0.10, post 5.46 ± 0.71, 24 h 1.88 ± 0.25, 48 h 0.46 ± 0.04; 5v5 rest 0.41 ± 0.07, post 4.07 ± 0.30, 24 h 2.41 ± 0.18, 48 h 0.45 ± 0.06	Soccer small-sided games: 1v1 and 5v5 formats, each totaling 24 min active play, performed in both phases	Salivary IL-6 and reactive strength index showed phase × format × time interactions after soccer small-sided games, with IL-6 generally higher in MLP, particularly after 1v1 sessions. DOMS did not show a significant phase × time interaction.
	MLP (days 20–23)	Not measured	Not measured	ML IL-6: 1v1 rest 1.23 ± 0.12, post 7.56 ± 0.32, 24 h 3.07 ± 0.30, 48 h 1.63 ± 0.37; 5v5 rest 1.25 ± 0.12, post 4.00 ± 0.55, 24 h 3.05 ± 0.24, 48 h 1.58 ± 0.20		
Yoshida et al. (27)	MP; days 1–5; measurement day 3.3 ± 0.7	37.7 ± 19.5	0.3 ± 0.1	Serum IL-6 and d-ROMs assessed pre-exercise, immediately post-exercise (Post0) and 60 min post-exercise (Post60)	60 min cycling at 65 ± 5% of age-predicted maximal heart rate	IL-6 increased immediately after moderate-intensity cycling, but menstrual phase was not the main explanatory factor for the response. d-ROMs showed no significant time or group effects.
	LP; latter half of cycle; measurement day 22.5 ± 3.4	168.4 ± 85.3	9.0 ± 6.8			

Creatine kinase or related muscle damage markers were reported in Funaki et al. (23), Graja et al. (17), Hackney et al. (26), Oosthuyse & Bosch (19), Romero-Parra et al. (21), Sipavičienė et al. (29), and Williams et al. (30). Funaki et al. (23) reported CK values before exercise and at 4 h, 48 h, and 96 h after eccentric elbow-flexor exercise; CK peaked at 96 h in both early follicular and mid-luteal groups, with no significant between-phase difference in the CK increase. Romero-Parra et al. (21) measured CK, myoglobin, LDH, CRP, TNF-α, and IL-6 before and after eccentric squat exercise and reported no significant menstrual-phase differences for CK, myoglobin, or LDH. Graja et al. (17) measured CK before and immediately after repeated sprint exercise and reported higher CK concentrations in the premenstrual phase than in the late follicular and mid-luteal phases. Hackney et al. (26) reported higher CK responses at 24 h and 72 h after prolonged treadmill running in the mid-follicular phase than in the mid-luteal phase. Williams et al. (30) reported higher total CK at 24 h after treadmill running in the mid-follicular condition than in the mid-luteal condition, while CK-MB did not differ by hormonal condition or time. Sipavičienė et al. (29) reported an increase in CK after stretch-shortening exercise without a significant difference between early follicular and late follicular/ovulatory testing. Oosthuyse & Bosch (19) measured CK and muscle soreness before, immediately after, and up to 72 h after downhill running; CK peaked at 24 h in the early follicular, late follicular, and mid-luteal groups, and pre-exercise CK did not differ between phase groups.

IL-6 or related inflammatory outcomes were reported in Alfaro-Magallanes et al. (15), Barba-Moreno et al. (16), Chaffin et al. (25), Gassner et al. (28), Hackney et al. (26), Minuzzi et al. (31), Romero-Parra et al. (21), Sawai et al. (22), Silva et al. (32), Yoshida et al. (27), and Zheng et al. (24). Chaffin et al. (25) reported an increase in IL-6 after interval running, with no significant menstrual-phase effect and no phase × time interaction. Sawai et al. (22) reported a significant time effect for IL-6 after moderate-intensity cycling, with a peak at 30 min post-exercise, but no significant main effect of phase and no statistically significant phase × time interaction. Minuzzi et al. (31) reported a time effect for IL-6 after high-intensity intermittent exercise, with lower values at 1 h post-exercise than at pre- and immediately post-exercise timepoints, and no phase × time interaction. Romero-Parra et al. (21) reported a time × phase interaction for IL-6, with higher 2 h post-exercise values in the mid-luteal phase than in early and late follicular phases. Hackney et al. (26) reported higher IL-6 responses in the mid-follicular phase than in the mid-luteal phase after prolonged treadmill running.

Barba-Moreno et al. (16) measured IL-6 and hepcidin before, immediately after, and 3 h after endurance running; IL-6 showed a menstrual phase × time interaction, with higher IL-6 at 3 h post-exercise in the luteal phase than in the early and mid-follicular phases, whereas hepcidin did not differ by menstrual phase. Alfaro-Magallanes et al. (15) measured hepcidin-25, IL-6, TNF-α, CRP, and iron-regulatory markers before and after interval running; hepcidin showed a time effect but no significant menstrual-phase effect or phase × time interaction, and IL-6 and TNF-α increased over time without a reported phase effect. Zheng et al. (24) measured IL-6 and hepcidin before, immediately after, and 3 h after cycling in moderate and warm-humid conditions; both IL-6 and hepcidin increased after exercise, but neither response differed by menstrual phase or ambient condition. Gassner et al. (28) measured ROS and IL-6 at baseline, pre-HIIT, post-HIIT, and 15 min post-HIIT and reported no significant differences in ROS or IL-6 responses by menstrual phase, estradiol group, or menstruation status. Silva et al. (32) measured salivary IL-6, DOMS, and reactive strength index after soccer small-sided games and reported significant phase × format × time interactions for IL-6 and reactive strength index, but not for DOMS. Yoshida et al. (27) measured serum IL-6 and d-ROMs before, immediately after, and 60 min after moderate-intensity cycling; IL-6 increased immediately after exercise, while d-ROMs showed no significant time or group effects.

Additional immune-cell or recovery-related outcomes were reported in Hicks et al. (18) and Parsons et al. (20). Hicks et al. (18) assessed peripheral blood mononuclear cell subsets before and immediately after a 5-km cycling time trial. Exercise-induced reductions in selected T-cell, NK-cell, and monocyte subsets were most evident during the early follicular phase and were attenuated or absent during the late follicular/ovulatory and mid-luteal phases. Parsons et al. (20) assessed hs-CRP across game-day recovery in recreational female athletes. Phase-specific model estimates showed a larger GD + 1 hs-CRP increase in the late luteal phase than in the early follicular, late follicular, or mid-luteal phases, with no significant phase-specific difference reported for GD + 2 or GD + 3.

Overall, 10 of 18 studies reported at least one menstrual phase-related difference or phase × time interaction for at least one eligible biomarker or recovery-related outcome, whereas eight studies reported predominantly time-dependent exercise responses without clear phase-related differences. However, the phase-related findings differed by biomarker, exercise model, endocrine contrast, and verification method, and did not form a consistent marker-specific pattern.

### 3.4 Methodological quality classification

Methodological quality classifications are summarized in Table 1 and Supplementary Tables S1A and S1B. Six studies retained a final high classification: Alfaro-Magallanes et al. (15), Funaki et al. (23), Graja et al. (17), Oosthuyse & Bosch (19), Romero-Parra et al. (21), and Zheng et al. (24). Four studies were classified as moderate: Barba-Moreno et al. (16), Hicks et al. (18), Sawai et al. (22), and Yoshida et al. (27). Eight studies were classified as low: Chaffin et al. (25), Gassner et al. (28), Hackney et al. (26), Minuzzi et al. (31), Parsons et al. (20), Silva et al. (32), Sipavičienė et al. (29), and Williams et al. (30). No included study was classified as very low.

The most common reasons for downgrading were absence of prospective ovulation testing and/or absence of blood-based confirmation of menstrual-cycle phase. Sampling schedules also differed by outcome type. Some studies included only immediate post-exercise sampling, whereas others included short-term sampling within 1–3 h or extended recovery sampling up to 24–96 h. These sampling designs are listed for each study in Table 3 and summarized together with methodological classification in Table 1.

## 4 Discussion

### 4.1 Main findings in relation to current menstrual cycle and exercise literature

This systematic review synthesized evidence from 18 studies examining whether menstrual cycle phase influences biomarkers or immune-cell outcomes related to exercise-induced muscle damage, inflammation, and acute post-exercise recovery responses in eumenorrheic or naturally menstruating women not using hormonal contraception. Across the included studies, the most consistent observation was that biomarker responses were strongly dependent on the exercise stimulus and the timing of post-exercise sampling. By contrast, menstrual phase-related differences were reported inconsistently and varied according to the biomarker assessed, the exercise model, the phase contrast, and the quality of menstrual phase verification.

This pattern is broadly consistent with the wider menstrual cycle and exercise literature. Previous systematic and narrative reviews have concluded that menstrual cycle phase may influence some exercise-related outcomes, but that group-level effects are generally small, inconsistent, and difficult to translate into universal recommendations for women or female athletes (1, 4, 7, 14). McNulty et al. (14), for example, reported only a trivial reduction in exercise performance during the early follicular phase compared with other phases, with large between-study variability and generally low-quality evidence. Similarly, reviews focused on elite athletes have emphasized that methodological heterogeneity and limited high-quality data currently preclude clear evidence-based recommendations for cycle phase-based training individualization (7). The present biomarker-focused review extends this broader conclusion to circulating and saliva-based markers of muscle damage, inflammation, and recovery: menstrual phase effects may occur in specific contexts, but the current evidence does not support a uniform or reproducible phase effect across biomarkers or exercise models.

For muscle damage-related outcomes, creatine kinase was the most frequently assessed marker. Some studies reported higher CK responses in lower-estrogen or premenstrual conditions, whereas others found no significant phase-related differences despite clear post-exercise increases. This inconsistency parallels the broader literature suggesting that menstrual cycle effects are unlikely to operate as simple phase-level main effects across all exercise contexts (1, 3). For inflammatory and immune-related outcomes, IL-6 was the most frequently measured marker and showed predominantly time-dependent responses after exercise. However, the direction of reported phase-related IL-6 effects was not uniform across studies. This finding is also consistent with the broader understanding that exercise-induced IL-6 responses are strongly shaped by exercise duration, intensity, muscle mass involved, metabolic stress, and sampling time, rather than by a single endocrine determinant (33, 34).

Additional outcomes, including hepcidin, hs-CRP, ROS, d-ROMs, PBMC subsets, salivary IL-6, DOMS, and reactive strength index, broadened the evidence base but were too heterogeneous to support marker-specific conclusions across menstrual phases. This reflects a more general challenge in female-specific sport and exercise science: interest in menstrual cycle-informed training and recovery has developed faster than the available biomarker evidence can currently support (2, 6, 35). Taken together, the present findings suggest that menstrual cycle phase should not be ignored in biomarker research, but neither should it be interpreted as a simple categorical modifier without rigorous endocrine verification and biomarker-specific sampling design.

### 4.2 Menstrual phase verification as a central methodological issue

The central methodological issue emerging from this review is the inconsistency with which menstrual cycle phases were defined and verified. This issue is widely recognized in the current menstrual cycle and sport science literature and is not merely a matter of reporting detail. The biological question concerns the potential influence of fluctuating ovarian hormones, particularly estradiol and progesterone, rather than the effect of calendar-defined cycle labels. Similar phase terms such as “follicular”, “luteal”, “mid-follicular”, or “mid-luteal” may therefore represent different endocrine conditions across studies if they are not supported by direct hormone assessment and ovulation confirmation.

Recent methodological papers have strongly cautioned against assuming or estimating menstrual cycle phases in laboratory and field-based sport research (2, 6). Elliott-Sale et al. (6) argue that assumed or estimated phases represent indirect approximations rather than direct measurements and should not be treated as equivalent to verified hormonal states. Calendar-based methods can identify bleeding days and approximate cycle timing, but they cannot reliably establish ovulation, luteal progesterone sufficiency, or the endocrine profile of non-menstruation phases. This distinction is particularly important because women may menstruate regularly while still presenting anovulatory or luteal phase-deficient cycles, and because ovulation timing and hormone concentrations vary substantially between individuals and between cycles (2, 6).

The laboratory medicine literature further supports this caution. Greene et al. (5) emphasize that estradiol, progesterone, LH, and FSH vary dynamically across the menstrual cycle and that interpretation of these hormones is analytically and biologically complex. Robust reference intervals remain incomplete, and random or poorly timed sampling can underestimate peak hormone concentrations or obscure meaningful endocrine variation (5). For exercise studies, this means that a nominal cycle phase does not necessarily indicate the hormonal exposure relevant to muscle damage, inflammation, substrate use, or recovery. Consequently, the absence of biochemical verification may reduce the ability to distinguish true hormone-related effects from phase misclassification.

The present review therefore distinguished between studies that verified menstrual phase using both blood-based ovarian hormone assessment and prospective ovulation testing, studies that included partial biochemical confirmation, and studies that relied on self-report, app-based tracking, or calendar estimation. This approach follows the logic applied by McNulty et al. (14), who incorporated menstrual phase verification into methodological quality appraisal by downgrading studies that did not confirm phase using blood samples or urinary ovulation testing. In the present review, this distinction directly affected the interpretive reliability of the evidence. Studies with stronger phase verification provide more confidence that observed biomarker differences, or absence of differences, were evaluated against biologically meaningful hormonal contrasts. By contrast, studies relying only on estimated cycle timing are more vulnerable to phase misclassification, which may dilute true hormone-related effects or create apparent phase differences that are difficult to interpret.

A further complication is that even verified studies did not always compare equivalent hormonal contrasts. Some studies compared early follicular and mid-luteal phases, others compared mid-follicular and mid-luteal phases, and several included late follicular, ovulatory, or premenstrual windows. These contrasts are not interchangeable. A low-hormone early follicular phase, a high-estradiol late follicular or ovulatory phase, and a high-estradiol/high-progesterone mid-luteal phase represent distinct endocrine environments (4, 5). Consequently, disagreement across studies should not be interpreted only as contradictory evidence; it may also reflect non-equivalent phase definitions, non-equivalent hormonal comparisons, and variable endocrine verification.

For future biomarker studies, menstrual phase terminology should therefore be linked explicitly to the verification method used. When only bleeding and cycle timing are documented, the term “naturally menstruating” may be more appropriate than “eumenorrheic”, and fine-grained phase labels should be avoided or clearly qualified (6). When the research question concerns hormone-mediated effects, biochemical confirmation of estradiol and progesterone, together with prospective ovulation testing where feasible, should be treated as a core design feature rather than as an optional refinement.

### 4.3 Creatine kinase and muscle damage-related markers: phase effects vs. biomarker kinetics

Creatine kinase was the most frequently assessed muscle damage-related marker in the included studies, but the evidence for menstrual phase-related modulation of CK was inconsistent. Studies using eccentric resistance or stretch-shortening protocols with delayed sampling generally reported clear time-dependent CK increases, but not consistent phase differences. Funaki et al. (23) found large CK elevations after eccentric elbow-flexor exercise, peaking at 96 h in both early follicular and mid-luteal groups, without a significant between-phase difference. Romero-Parra et al. (21) similarly reported time-dependent changes in CK and related markers after eccentric squat exercise, with no meaningful phase differences across early follicular, late follicular, and mid-luteal phases. Sipavičienė et al. (29) observed CK increases after stretch-shortening exercise but no significant difference between early follicular and late follicular/ovulatory testing.

These findings are consistent with the broader EIMD literature, which emphasizes that CK is a delayed, indirect, and highly variable marker of muscle membrane disruption rather than a direct measure of local structural damage. Exercise-induced muscle damage is most strongly associated with unaccustomed or high-intensity eccentric work, sarcomere disruption, altered excitation-contraction coupling, calcium dysregulation, and subsequent inflammatory and remodeling processes (9–11, 36). Circulating CK appears only after intracellular proteins leak into the extracellular space and circulation, and its time course often extends beyond the immediate post-exercise period. Therefore, studies that sample CK only immediately after exercise are poorly aligned with its expected kinetics. This is particularly relevant for Graja et al. (17), where CK was measured only before and immediately after repeated sprint exercise. Although higher CK concentrations were reported in the premenstrual phase, the sampling schedule could not capture the delayed CK peak typically expected after damaging exercise.

The delayed nature and biological variability of CK may also explain why phase-related findings differed across studies. Brancaccio et al. (37) emphasized that CK responses depend on multiple factors, including sex, muscle mass, training status, physical activity history, environmental conditions, exercise type, and individual responder characteristics. These factors are highly relevant to the present review because the included studies ranged from sedentary women to trained runners, handball players, endurance-trained women, soccer players, and recreational athletes. In small samples, inter-individual variability in CK release and clearance may be large enough to obscure modest menstrual phase-related effects, even when endocrine contrasts are biologically meaningful.

Some endurance-based studies reported higher CK responses in lower-estradiol conditions. Hackney et al. (26) found higher CK responses at 24 h and 72 h after prolonged treadmill running in the mid-follicular phase than in the mid-luteal phase, and Williams et al. (30) reported higher total CK at 24 h in the mid-follicular condition than in the mid-luteal condition. These findings are compatible with the hypothesis that estradiol-related phenolic structures may contribute to antioxidant protection and reduced lipid peroxidation, but direct extrapolation to exercise-induced muscle damage should remain cautious because this mechanism has not been consistently demonstrated in menstrual cycle exercise studies (3, 13). In addition, Williams et al. (30) verified estradiol but not progesterone or ovulation, and Hackney et al. (26) included a small pilot sample without prospective ovulation testing. Moreover, CK-MB did not differ by phase in Williams et al. (30), suggesting that phase-related effects may not generalize across related muscle enzyme markers.

The pattern across studies therefore does not support a simple protective-estrogen model. If estradiol exerts membrane-stabilizing or antioxidant effects, these effects may be most visible in specific exercise contexts, particularly where oxidative stress and membrane permeability contribute materially to biomarker release. By contrast, in protocols involving a large eccentric mechanical load, the magnitude of structural disruption may dominate the biomarker response and reduce the detectability of smaller endocrine effects. This interpretation is consistent with contemporary mechanistic reviews, which frame EIMD as a multifactorial process involving mechanical strain, calcium handling, oxidative stress, inflammation, remodeling, and prior exposure rather than a single pathway (9, 11, 36).

A further issue is the repeated-bout effect. Prior exposure to eccentric or damaging exercise can markedly attenuate symptoms, strength loss, and CK responses during subsequent bouts (9, 10, 36). This is critical for menstrual cycle studies using repeated within-subject exercise trials across phases. If trial order, familiarization, washout duration, and recent training load are not carefully controlled, apparent phase differences may be confounded by adaptation to the exercise stimulus. Conversely, true phase-related effects may be masked by protective adaptation from the first trial. Future studies should therefore report trial order, familiarization procedures, washout periods, prior damaging exercise, and training load in sufficient detail.

Overall, CK remains useful for characterizing systemic leakage of muscle-derived enzymes after exercise, but its interpretation in menstrual cycle research requires biomarker-specific sampling and strong control of confounders. The current evidence indicates that CK responses are clearly affected by exercise and recovery time, whereas menstrual phase effects are inconsistent and likely depend on the interaction between endocrine milieu, exercise modality, damage severity, sampling schedule, and individual responder profile. Evidence for other muscle damage-related markers, including LDH and myoglobin, remains too limited to determine whether they are meaningfully modulated by menstrual phase.

### 4.4 IL-6 and inflammatory markers: exercise response, myokine biology, and menstrual phase

IL-6 was the most frequently measured inflammatory marker in the included studies, but the direction of menstrual phase-related effects was not consistent. Several studies reported predominantly time-dependent IL-6 responses without significant phase effects. Chaffin et al. (25) found that IL-6 increased after interval running, with no significant menstrual phase effect or phase × time interaction. Sawai et al. (22) reported a clear post-exercise IL-6 rise after moderate-intensity cycling, peaking at 30 min, but no significant main effect of phase and no statistically significant phase × time interaction. Minuzzi et al. (31) also reported a time effect after high-intensity intermittent exercise, but no phase × time interaction.

These findings align with the broader exercise immunology literature, in which IL-6 is understood not only as an inflammatory cytokine but also as a contraction-induced myokine. Muscle-derived IL-6 can rise rapidly during and after exercise, with the magnitude of increase shaped by exercise duration, intensity, muscle mass involved, training status, glycogen availability, and metabolic stress (33, 34). Importantly, an acute exercise-induced IL-6 response does not necessarily indicate muscle damage or harmful inflammation. Rather, IL-6 participates in metabolic regulation, substrate mobilization, and anti-inflammatory signaling, including stimulation of IL-1 receptor antagonist and IL-10 and suppression of TNF-α production in some contexts (33, 34). This distinction is important for the present review because IL-6 cannot be interpreted as a simple proxy for EIMD severity.

The included studies reporting phase-related IL-6 effects differed in both direction and context. Hackney et al. (26) reported higher IL-6 responses after prolonged treadmill running in the mid-follicular phase than in the mid-luteal phase. In contrast, Romero-Parra et al. (21) reported a time × phase interaction after eccentric squat exercise, with higher IL-6 at 2 h post-exercise in the mid-luteal phase than in early or late follicular phases. Barba-Moreno et al. (16) similarly observed higher IL-6 at 3 h post-exercise in the luteal phase after endurance running, whereas Zheng et al. (24) and Alfaro-Magallanes et al. (15) found post-exercise IL-6 increases without a clear phase effect. Gassner et al. (28) and Yoshida et al. (27) also reported acute IL-6 responses that were primarily time- or workload-related rather than clearly phase-dependent. Silva et al. (32), using salivary IL-6 after soccer small-sided games, found phase × format × time interactions, but the use of calendar-based phase classification and saliva as the biological matrix limits comparability with blood-based studies.

This mixed pattern suggests that menstrual phase, if relevant to IL-6 responses, is unlikely to act as a uniform categorical determinant. Instead, phase effects may depend on the interaction between endocrine milieu and the specific physiological stressor. Prolonged endurance exercise, repeated high-intensity exercise, eccentric resistance exercise, small-sided games, and moderate cycling differ substantially in metabolic demand, mechanical strain, muscle mass recruitment, glycogen use, and inflammatory stimulus. These factors are all known to affect IL-6 kinetics (33, 34). Therefore, inconsistent phase-related IL-6 findings may reflect heterogeneity in exercise physiology as much as heterogeneity in menstrual phase verification.

Sampling design is also critical. IL-6 typically rises rapidly and may peak at or shortly after the end of exercise, followed by a decline toward baseline during recovery. Studies using only a single immediate post-exercise sample may capture part of this response, whereas studies sampling at 2–3 h may emphasize a different phase of recovery. This matters because some of the reported phase differences occurred at later early-recovery timepoints, such as 2 h or 3 h post-exercise (16, 21), whereas other studies emphasized immediate or 30–60 min responses (22, 25, 27). Without dense early sampling, it is difficult to determine whether phase affects the peak IL-6 response, the timing of the peak, or the rate of return toward baseline.

The broader menstrual-cycle immunology literature also supports caution in interpreting cytokine responses as simple hormone-driven effects. Baseline cytokine and immune profiles across the cycle appear cytokine-specific and cell-type-specific: Brännström et al. (38) reported cyclic variation in TNF-α but not IL-6, Faas et al. (39) found evidence for a luteal shift toward an IL-4-related Th2-type response, Maskill et al. (40) reported stable serum IL-10 across the cycle, and O'Brien et al. (41) observed higher luteal IL-4, TNF-α, and sIL-6R but no cycle variation in IL-6, IL-8, or IL-10. Bouman et al. (42) further showed that estradiol and progesterone did not directly alter the cytokine-producing capacity of LPS-stimulated monocytes for TNF-α and IL-1β. Together, these findings argue against a simple model in which ovarian hormones uniformly increase or suppress systemic inflammation. This distinction is consistent with the present review, in which Minuzzi et al. (31) reported higher resting TNF-α in the luteal phase, whereas exercise-induced cytokine changes were primarily time-dependent.

Overall, the available evidence supports IL-6 as a robust exercise-responsive marker, but not as a consistent menstrual phase marker. Future studies should treat IL-6 as a dynamic myokine and immune-metabolic signal rather than as a static inflammatory endpoint. This requires blood-based sampling schedules that capture early post-exercise kinetics, clear documentation of exercise intensity and duration, dietary and glycogen-related controls where possible, and rigorous menstrual phase verification. Such designs are needed to determine whether ovarian hormone fluctuations influence IL-6 amplitude, timing, recovery slope, or baseline concentration in a way that is biologically meaningful for exercise recovery.

### 4.5 Emerging biomarkers and recovery-related outcomes

Beyond CK and IL-6, the included studies assessed several emerging or recovery-related outcomes, including hepcidin and iron-regulatory markers, hs-CRP, ROS, d-ROMs, PBMC subsets, salivary IL-6, DOMS, and reactive strength index. These outcomes expand the scope of menstrual cycle biomarker research, but they also introduce additional interpretive complexity because they do not represent a single physiological domain. Some are more closely related to inflammation, others to iron regulation, oxidative stress, immune-cell redistribution, subjective soreness, or neuromuscular recovery. Therefore, they should not be interpreted as interchangeable indicators of exercise-induced muscle damage.

Hepcidin and iron-regulatory outcomes were assessed in endurance-based protocols by Alfaro-Magallanes et al. (15), Barba-Moreno et al. (16), and Zheng et al. (24). These studies are relevant because IL-6 is a known upstream signal involved in post-exercise hepcidin regulation, and because iron availability is particularly important in female athletes. However, the findings did not indicate a uniform menstrual phase effect on hepcidin. Barba-Moreno et al. (16) reported no phase difference in hepcidin despite a phase × time interaction for IL-6, whereas Alfaro-Magallanes et al. (15) found a time effect for hepcidin but no significant phase or phase × time interaction. Zheng et al. (24) similarly reported post-exercise increases in IL-6 and hepcidin that were not affected by menstrual phase or ambient condition. These studies suggest that hepcidin may be a useful marker for cycle-aware exercise research, but its interpretation belongs primarily to iron regulation and immune-metabolic response rather than to direct EIMD assessment.

CRP-related evidence was extended by Parsons et al. (20), who examined hs-CRP across game-day recovery in recreational female athletes. This field-based design is valuable because it reflects sport exposure under ecologically relevant conditions and used repeated recovery sampling across several days. The larger GD + 1 hs-CRP increase reported in the late luteal phase may indicate a phase-related recovery signal. However, hs-CRP is a delayed systemic acute-phase marker, and field-based game exposure introduces variability in exercise dose, contact load, fatigue, injury, illness, and behavioral recovery. In addition, menstrual phase classification did not include blood-based estradiol or progesterone confirmation. Consequently, this study is useful for hypothesis generation but cannot establish a direct causal link between ovarian hormone milieu and CRP recovery.

Oxidative stress-related outcomes were addressed in Gassner et al. (28) and Yoshida et al. (27). Gassner et al. (28) measured ROS and IL-6 responses to resistance circuit HIIT and found no significant differences by menstrual phase, estradiol group, or menstruation status. Yoshida et al. (27) measured d-ROMs alongside IL-6 after moderate-intensity cycling and found no significant time or group effects for d-ROMs. These findings are relevant because estradiol has been proposed to influence antioxidant protection and membrane stability (3, 13). However, current exercise studies do not yet provide consistent evidence that menstrual phase meaningfully modifies systemic oxidative stress markers after acute exercise. This may reflect true absence of a large effect, but it may also reflect limitations in marker specificity, sampling timing, exercise stimulus, and endocrine verification.

Immune-cell redistribution was explored by Hicks et al. (18), who assessed PBMC subsets before and immediately after a 5-km cycling time trial. The observed phase-related differences in selected T-cell, NK-cell, and monocyte subsets are notable because they move beyond soluble biomarkers and address cellular immune responses. However, immune-cell redistribution during acute exercise is strongly influenced by catecholamines, cortisol, exercise intensity, and timing of blood sampling. Therefore, these results should be interpreted as exploratory evidence that menstrual cycle phase may influence acute immune-cell mobilization, rather than as direct evidence of altered inflammatory recovery or muscle damage. Larger studies with dense sampling and stronger endocrine verification are needed to determine whether these cell-specific patterns are reproducible.

Applied recovery outcomes were assessed most directly by Silva et al. (32), who measured salivary IL-6, DOMS, and reactive strength index after soccer small-sided games. This study is valuable because it links inflammatory, subjective, and performance-related recovery outcomes in an applied sport setting. However, salivary IL-6 is not directly interchangeable with serum or plasma IL-6, and DOMS and reactive strength index reflect different dimensions of recovery. DOMS is subjective and does not necessarily parallel circulating CK or cytokine responses, while reactive strength index captures neuromuscular performance rather than inflammation alone. The calendar-based phase classification also limits endocrine interpretation. These findings therefore support the inclusion of multi-domain recovery outcomes in future studies, but also highlight the need to distinguish biological matrix, subjective symptoms, and functional recovery when interpreting menstrual phase effects.

Overall, emerging biomarkers and recovery-related outcomes offer important opportunities for future research, but the current evidence remains too heterogeneous for firm conclusions. Future studies should define in advance whether the primary target is muscle membrane disruption, inflammatory signaling, immune-cell redistribution, oxidative stress, iron regulation, subjective recovery, or neuromuscular performance. Without this conceptual separation, there is a risk of combining biologically distinct markers under the broad label of “inflammation” or “recovery”, thereby weakening interpretation of menstrual phase effects.

### 4.6 Methodological considerations and evidence gaps

The evidence base remains limited by recurring methodological issues that cluster around menstrual phase verification, exercise-protocol heterogeneity, biomarker-specific sampling, confounder control, and statistical design. These issues are not independent: weak endocrine verification reduces confidence in phase attribution, while heterogeneous exercise stimuli and poorly aligned sampling schedules reduce confidence in biomarker interpretation.

Another major limitation is heterogeneity of exercise protocols. The included studies used eccentric elbow-flexor exercise, eccentric squat exercise, stretch-shortening exercise, downhill running, prolonged treadmill running, repeated sprint exercise, high-intensity intermittent exercise, moderate cycling, HIIT, cycling time trials, soccer small-sided games, and field-based game-day exposure. These models differ substantially in mechanical strain, eccentric loading, metabolic demand, muscle mass recruited, glycogen use, and inflammatory stimulus. Such heterogeneity makes it difficult to determine whether inconsistent phase effects reflect true biological variability or simply different exercise stimuli. This is particularly important because CK, IL-6, CRP, hepcidin, ROS, immune-cell redistribution, DOMS, and performance-related recovery outcomes are each expected to respond differently to these stimuli.

Another limitation is the mismatch between biomarker kinetics and sampling design in some studies. CK and CRP require delayed recovery sampling, whereas IL-6 and some immune-cell responses require dense early sampling. Oxidative stress markers and hepcidin may require intermediate timepoints, and subjective or neuromuscular recovery outcomes may evolve across 24–72 h. Studies that sample only immediately post-exercise cannot adequately characterize delayed muscle damage markers, whereas studies lacking early post-exercise samples may miss peak IL-6 or acute immune-cell redistribution. Future studies should therefore design sampling schedules around the expected kinetics of each biomarker rather than applying a uniform time schedule across all outcomes.

Another limitation is control of confounding variables. Biomarker responses to exercise are influenced by prior exercise, training status, habitual activity, body composition, adiposity, sleep, nutrition, carbohydrate availability, hydration, alcohol and caffeine intake, anti-inflammatory medication, illness, injury, psychological stress, time of day, and environmental conditions. Some included studies controlled these factors carefully, but others reported them incompletely. Because many of these variables may interact with both menstrual cycle physiology and exercise recovery, incomplete reporting can substantially reduce interpretability.

Another limitation concerns study design and statistical power. Most studies included small samples, and several were exploratory or pilot in nature. Small samples are particularly problematic for biomarkers such as CK and cytokines, which show large inter-individual variability. Within-subject designs can reduce inter-individual variability, but they introduce the risk of repeated-bout effects when damaging exercise protocols are repeated across phases. Between-group phase designs avoid repeated-bout adaptation but are more vulnerable to between-person variability in baseline biomarker concentrations, training status, and endocrine profiles. Future studies should justify sample size, use appropriate repeated-measures or mixed-effects models, report effect sizes and confidence intervals, and distinguish within-person phase effects from between-person variability.

A further gap is the limited integration of biomarkers with functional and subjective recovery outcomes. Most studies focused on circulating or saliva-based markers, while fewer included performance recovery, soreness, immune-cell function, or athlete-reported symptoms. Yet menstrual cycle-related changes may be more apparent in subjective or functional domains than in circulating biomarkers alone. Conversely, biomarker changes may occur without perceptible performance or recovery consequences. Future research should therefore integrate endocrine verification, biomarker kinetics, performance outcomes, and symptom tracking within the same study design.

Finally, the current evidence does not support simple menstrual phase-based biomarker rules for sport practice. The more appropriate conclusion is that menstrual status should be treated as a biologically relevant design and interpretation variable. This means that researchers should measure and report menstrual status rigorously, but should avoid assuming that phase labels alone explain biomarker variability. The next generation of studies should move from descriptive phase comparisons toward analytically explicit models that test whether estradiol, progesterone, ovulation status, or symptom profiles explain meaningful within-person variability in exercise recovery biomarkers.

### 4.7 Recommendations for future analytical design

Future studies should move from nominal menstrual phase comparisons toward analytically explicit, cycle-aware biomarker designs. The first requirement is precise definition of the physiological question. Studies should specify whether they aim to test effects of menstrual phase labels, measured estradiol and progesterone concentrations, ovulation status, symptom burden, or within-person hormonal change. These are related but distinct research questions. Treating menstrual phase as a simple categorical variable may be insufficient when the underlying mechanism is presumed to involve continuous or threshold-dependent hormone exposure.

A minimum recommended approach should include prospective cycle tracking, confirmation of ovulation where feasible, and blood-based assessment of estradiol and progesterone at each experimental trial. For studies claiming early follicular, late follicular/ovulatory, and mid-luteal comparisons, urinary LH testing provides an important physiological anchor for ovulation timing, but should not be treated as full endocrine verification of phase-specific hormone exposure unless supported by estradiol and progesterone concentrations compatible with the intended phase. Conversely, when only bleeding onset and calendar timing are available, authors should avoid over-specific phase terminology and clearly state that phase classification was estimated. Reporting should include the method of phase allocation, cycle day, ovulation verification procedure, actual estradiol and progesterone values, and criteria used to exclude anovulatory or luteal phase-deficient cycles.

The exercise protocol should be selected according to the biomarker domain of interest. If the primary outcome is EIMD, the protocol should include a sufficiently damaging and reproducible mechanical stimulus, preferably with a clearly defined eccentric component, standardized range of motion, workload, and recovery period. If the primary outcome is IL-6 or immune-metabolic signaling, exercise intensity, duration, muscle mass involved, glycogen-related controls, and timing of early post-exercise sampling should be prioritized. If the study focuses on hepcidin or iron regulation, iron status, recent dietary iron intake, inflammation, and the IL-6-to-hepcidin temporal window should be incorporated into the design. If the focus is applied recovery, biomarkers should be integrated with functional and subjective recovery outcomes rather than interpreted in isolation.

Sampling schedules should be biomarker-specific. CK, myoglobin, LDH, and CRP require delayed recovery sampling, often extending at least 24–72 h and, for CK, preferably up to 96 h when severe EIMD is expected. IL-6 requires dense early sampling, including immediately post-exercise and additional samples within the first 30–180 min, depending on exercise duration and intensity. Hepcidin should include a 3 h post-exercise window, while immune-cell redistribution requires immediate and short-term post-exercise timepoints. DOMS and neuromuscular recovery outcomes should be followed for at least 24–72 h. Studies using a single universal sampling schedule across all biomarkers risk missing the physiologically relevant response window for some outcomes.

Future studies should also control and report confounding variables more consistently. At minimum, authors should document prior exercise, recent muscle-damaging activity, training status, diet, carbohydrate availability, fasting state, hydration, alcohol and caffeine intake, anti-inflammatory medication, sleep, illness or injury, time of day, and environmental conditions. For within-subject designs, trial order should be randomized or counterbalanced, and washout periods should be long enough to reduce repeated-bout effects. Familiarization procedures should be described clearly, especially when the exercise protocol itself may induce protective adaptation.

Statistical analysis should reflect the repeated, multilevel nature of menstrual cycle biomarker data. Mixed-effects models are generally more appropriate than simple phase comparisons because they can account for within-person clustering, time effects, phase × time interactions, and relevant covariates. Studies should report effect sizes, confidence intervals, individual response patterns, and raw or minimally processed biomarker values wherever possible. Because biomarker responses are often skewed and highly variable, pre-specified handling of outliers, transformations, and responder/non-responder analyses should also be reported. Importantly, absence of statistical significance should not be interpreted as evidence of no biological effect when sample sizes are small and phase verification is incomplete.

Finally, future research should move beyond broad phase labels toward integrated models combining endocrine, biomarker, performance, and symptom data. Such designs would allow investigators to test whether measured estradiol, progesterone, their ratio, ovulation status, or symptom burden explain within-person variation in post-exercise biomarker responses. This direction is also supported by molecular exercise biomarker research showing that sex-specific and menstrual cycle-aware analyses may reveal biomarker-performance relationships that are obscured or misinterpreted when women and men are analyzed together (43). Integrated endocrine-biomarker models are more likely to generate biologically meaningful evidence than comparisons of broad menstrual phase categories across heterogeneous exercise protocols.

### 4.8 Practical implications

The present findings do not support broad practical recommendations to modify training or recovery strategies solely based on menstrual cycle phase in relation to circulating biomarkers of muscle damage or inflammation. Although some studies reported phase-related biomarker differences, the direction and magnitude of these effects were not consistent across biomarkers, exercise modalities, or sampling schedules. Therefore, current evidence is insufficient to conclude that a specific menstrual phase is uniformly associated with greater muscle damage, stronger inflammation, or impaired biomarker recovery.

This does not mean that menstrual status is irrelevant for athletes or exercise research. Rather, menstrual status should be treated as one component of an individualized monitoring framework. In practice, symptoms, perceived recovery, sleep, mood, soreness, training load, performance, nutrition, and injury or illness status may provide more directly actionable information than menstrual phase labels alone. Biomarkers may add value when interpreted in this broader context, but isolated CK, IL-6, CRP, or other marker values should not be overinterpreted without considering exercise exposure, sampling time, prior training, and biological variability.

For researchers and practitioners, the most appropriate current implication is therefore not cycle phase-based prescription, but cycle-aware interpretation. Menstrual cycle information should be documented, and where possible verified, when collecting biomarkers from naturally menstruating women. This is particularly important in longitudinal monitoring, intervention studies, and studies comparing female and male athletes, because unrecorded menstrual status may contribute to unexplained variability. However, the menstrual cycle phase should not be treated as a deterministic explanation for biomarker changes in the absence of endocrine confirmation and appropriate sampling design. This interpretation is consistent with recent work showing that menstrual cycle-related changes in physical and psychological parameters may be detectable at the group level, but remain sufficiently individual to support pattern-oriented and individually adaptive monitoring rather than rigid phase-based training prescriptions (44).

A realistic promise for future applied work is the development of individualized recovery profiles that integrate menstrual status with symptoms, workload, and biomarker trajectories. Such an approach would be more consistent with current evidence than universal phase-based rules. It would also reflect the fact that women may differ substantially in hormone concentrations, cycle regularity, symptom burden, training history, and biomarker responsiveness. Until stronger evidence is available, menstrual cycle tracking should be used to contextualize athlete monitoring rather than to impose fixed assumptions about inflammation or muscle damage.

### 4.9 Strengths and limitations of the review

A major strength of this review is its focused synthesis of studies examining exercise-induced muscle damage, inflammation, immune-cell outcomes, and recovery-related biomarkers across menstrual cycle phases in naturally menstruating women not using hormonal contraception. The review used a systematic search strategy across PubMed, Scopus, and Web of Science Core Collection, applied predefined eligibility criteria, and incorporated a structured methodological quality appraisal. By distinguishing *a priori* methodological quality from menstrual phase verification downgrades, the review specifically addressed a central limitation in menstrual cycle exercise research.

Another strength is the integration of biomarker findings with methodological interpretation. Rather than treating all outcomes as equivalent inflammatory markers, the review separated muscle damage-related enzymes, IL-6 and inflammatory cytokines, hepcidin and iron-regulatory outcomes, oxidative stress markers, immune-cell subsets, hs-CRP, subjective soreness, and neuromuscular recovery outcomes. This distinction is important because each marker reflects a different physiological process and has a different expected time course after exercise.

Several limitations should also be acknowledged. First, the evidence base was heterogeneous in participant characteristics, training status, menstrual phase definitions, phase verification procedures, exercise protocols, biological matrices, biomarker panels, and sampling schedules. This heterogeneity precluded meta-analysis and also limited the applicability of formal outcome-level GRADE assessment. We therefore did not grade certainty separately for each outcome; instead, the strength of the evidence was addressed through study-level methodological appraisal, predefined menstrual phase verification downgrades, and a review-specific interpretive reliability summary. Second, many included studies had small sample sizes, and several were exploratory or pilot studies. As a result, both false-negative and false-positive phase effects are possible. Third, not all studies included rigorous endocrine verification, and some relied on calendar- or app-based phase estimation. This limits confidence in phase-specific interpretation. Fourth, the included outcomes extended beyond classic EIMD biomarkers to adjacent immune, oxidative stress, iron-regulatory, and recovery-related outcomes. This broadened the relevance of the review but also increased conceptual heterogeneity. Fifth, although the review focused on women not using hormonal contraception, reporting of hormonal contraceptive exclusion, medication use, illness, prior exercise, nutrition, sleep, and recovery behavior varied across studies. Finally, because most studies reported group-level phase comparisons, the review could not determine whether individual women show reproducible within-person biomarker patterns across cycles.

Despite these limitations, the review identifies a consistent conclusion: exercise stimulus and sampling time are more reproducible determinants of biomarker responses than menstrual phase labels alone. The current evidence supports the need for better designed, cycle-aware biomarker studies rather than immediate translation into universal phase-based training or recovery recommendations.

## 5 Conclusion

This systematic review synthesized evidence on biomarkers, immune-cell outcomes, and recovery-related responses to acute exercise across menstrual cycle phases in naturally menstruating women not using hormonal contraception. Across 18 included studies, exercise stimulus and post-exercise sampling time emerged as more consistent determinants of biomarker responses than menstrual phase labels alone. Clear exercise-related changes were observed for several outcomes, particularly CK and IL-6, but menstrual phase-related differences were inconsistent across biomarkers, exercise models, endocrine contrasts, and sampling schedules.

The current evidence does not support a uniform menstrual phase effect on biomarkers of exercise-induced muscle damage or inflammation. Where phase-related differences were reported, their interpretation was limited by small samples, heterogeneous protocols, incomplete menstrual phase verification, non-equivalent hormonal comparisons, and biomarker sampling schedules that were not always aligned with expected response kinetics. Therefore, menstrual cycle phase should be treated as a biologically relevant design and interpretation variable, but not as a deterministic explanation for post-exercise biomarker variation.

Future research should prioritize cycle-aware study designs that combine prospective cycle tracking, ovulation confirmation, blood-based estradiol and progesterone assessment, standardized exercise stimuli, biomarker-specific sampling schedules, and appropriate repeated-measures or mixed-effects statistical models. Such approaches are needed to determine whether ovarian hormone fluctuations meaningfully explain within-person variability in post-exercise biomarker responses and to support more precise, biologically informed research in female exercise physiology.
