# Supplementary material for: Biomarkers of exercise-induced muscle damage and inflammation across menstrual cycle phases: a critical review
Source: Front Sports Act Living. 2026 Jul 30;8:1878668. doi: 10.3389/fspor.2026.1878668 (PMC13471081; doi:10.3389/fspor.2026.1878668)
Supplement: Supplementary file 1 [file Table1.docx]

Supplementary Material

**Search Strategy**

The search strategy combined terms related to menstrual cycle status, exercise, muscle damage, inflammation, and circulating biomarkers. Searches were performed in PubMed, Web of Science Core Collection, and Scopus. The database searches were not restricted by language or document type at the search stage, because language and document-type metadata may be incomplete or inconsistently assigned across databases. Eligibility restrictions, including English full-text availability, original research design, regular naturally occurring menstrual cycles, and absence of hormonal contraceptive use, were applied during title/abstract and full-text screening. Reviews and other non-original articles were not included in the qualitative synthesis.

**Web of Science Core Collection n = 428**

Search mode: Advanced Search, Query Builder

Field: Topic (TS)

Search period: database inception to June 21, 2026

Exact Search: enabled

Document types: Article; Early Access

Language restriction: none

TS = (

("menstrual cycle" OR "menstrual phase*" OR "cycle phase*" OR eumenorrheic OR eumenorrhoeic OR "follicular phase" OR "luteal phase" OR "ovulatory phase" OR ((follicular OR luteal OR ovulatory) NEAR/3 (phase* OR cycle*)))

AND

(exercise OR "physical activity" OR sport* OR training OR running OR cycling OR endurance OR resistance OR strength OR eccentric OR sprint* OR "high-intensity interval" OR HIIT OR HIIE)

AND

("muscle damage" OR "exercise-induced muscle damage" OR EIMD OR "muscle soreness" OR DOMS OR cytokine* OR leukocyte* OR neutrophil* OR "creatine kinase" OR CK OR myoglobin OR "lactate dehydrogenase" OR LDH OR "C-reactive protein" OR CRP OR hs-CRP OR hsCRP OR "high-sensitivity C-reactive protein" OR "interleukin-6" OR IL-6 OR IL6 OR TNF OR "tumor necrosis factor" OR "tumour necrosis factor")

)

**PubMed n = 288**

Search mode: Advanced Search

Field: Title/Abstract ([tiab])

Search period: database inception to June 21, 2026

Language restriction: none

Article-type restriction: none

Animal-only studies excluded using: NOT (animals[mh] NOT humans[mh])

(

("menstrual cycle"[tiab] OR "menstrual cycles"[tiab] OR "menstrual phase"[tiab] OR "menstrual phases"[tiab] OR "cycle phase"[tiab] OR "cycle phases"[tiab] OR eumenorrheic[tiab] OR eumenorrhoeic[tiab] OR "follicular phase"[tiab] OR "luteal phase"[tiab] OR "ovulatory phase"[tiab] OR ((follicular[tiab] OR luteal[tiab] OR ovulatory[tiab]) AND (phase*[tiab] OR cycle*[tiab])))

AND

(exercise[tiab] OR "physical activity"[tiab] OR sport*[tiab] OR training[tiab] OR running[tiab] OR cycling[tiab] OR endurance[tiab] OR resistance[tiab] OR strength[tiab] OR eccentric[tiab] OR sprint*[tiab] OR "high-intensity interval"[tiab] OR HIIT[tiab] OR HIIE[tiab])

AND

("muscle damage"[tiab] OR "exercise-induced muscle damage"[tiab] OR EIMD[tiab] OR "muscle soreness"[tiab] OR DOMS[tiab] OR cytokine*[tiab] OR leukocyte*[tiab] OR neutrophil*[tiab] OR "creatine kinase"[tiab] OR CK[tiab] OR myoglobin[tiab] OR "lactate dehydrogenase"[tiab] OR LDH[tiab] OR "C-reactive protein"[tiab] OR CRP[tiab] OR hs-CRP[tiab] OR hsCRP[tiab] OR "high-sensitivity C-reactive protein"[tiab] OR "interleukin-6"[tiab] OR IL-6[tiab] OR IL6[tiab] OR TNF[tiab] OR "tumor necrosis factor"[tiab] OR "tumour necrosis factor"[tiab])

)

NOT

(animals[mh] NOT humans[mh])

**Scopus n = 710**

Search mode: Advanced document search

Field: Title, Abstract, Keywords (TITLE-ABS-KEY)

Search period: database inception to June 21, 2026

Document types: Article; Article in Press

Language restriction: none

TITLE-ABS-KEY (

("menstrual cycle" OR "menstrual phase" OR "menstrual phases" OR "cycle phase" OR "cycle phases" OR eumenorrheic OR eumenorrhoeic OR "follicular phase" OR "luteal phase" OR "ovulatory phase" OR ((follicular OR luteal OR ovulatory) W/3 (phase* OR cycle*)))

AND

(exercise OR "physical activity" OR sport* OR training OR running OR cycling OR endurance OR resistance OR strength OR eccentric OR sprint* OR "high-intensity interval" OR HIIT OR HIIE)

AND

("muscle damage" OR "exercise-induced muscle damage" OR EIMD OR "muscle soreness" OR DOMS OR cytokine* OR leukocyte* OR neutrophil* OR "creatine kinase" OR CK OR myoglobin OR "lactate dehydrogenase" OR LDH OR "C-reactive protein" OR CRP OR "hs-CRP" OR hsCRP OR "high-sensitivity C-reactive protein" OR "interleukin-6" OR "IL-6" OR IL6 OR TNF OR "tumor necrosis factor" OR "tumour necrosis factor")

)

AND PUBYEAR < 2027

AND (DOCTYPE(ar) OR DOCTYPE(ip))

**Population, intervention, comparator, outcomes and study design (PICOS) criteria**

| Population | Eumenorrheic women of reproductive age with regular, naturally occurring menstrual cycles and no use of hormonal contraception. |
| --- | --- |
| Intervention | An acute exercise bout or sport-related exercise exposure expected to induce muscle damage and/or an inflammatory response. |
| Comparator | Comparison of biomarker responses across two or more menstrual cycle phases within the same study. |
| Outcomes | Blood- or saliva-derived biomarkers, circulating immune-cell outcomes, or closely related recovery markers relevant to exercise-induced muscle damage, inflammation, acute immune response, oxidative stress, iron regulation, or recovery. Eligible outcomes included, but were not limited to, creatine kinase, myoglobin, lactate dehydrogenase, C-reactive protein, high-sensitivity C-reactive protein, interleukin-6, tumor necrosis factor-α, hepcidin, oxidative-stress markers, immune-cell subsets, delayed-onset muscle soreness, and neuromuscular recovery-related outcomes. |
| Study design | Original peer-reviewed studies using within-subject or phase-based comparisons across menstrual cycle phases and reporting extractable biomarker data. |

**Table S1A.** Modified Downs and Black appraisal items and scoring rules

| **Item** | **Appraisal criterion** | **Scoring rule** |
| --- | --- | --- |
| Q1 | Is the hypothesis/aim/objective of the study clearly described? | Yes = 1 / No = 0 |
| Q2 | Are the main outcomes to be measured clearly described in the introduction or methods section? If the main outcomes are first mentioned in the results section, answer No. | Yes = 1 / No = 0 |
| Q3 | Are the characteristics of the participants included in the study clearly described? | Yes = 1 / No = 0 / Unable to determine = 0 |
| Q4 | Were the tested menstrual cycle phases clearly described? Answer Yes if the precise criteria used to define phase were provided, answer No if the exact phase tested cannot be ascertained (e.g., vague language such as “early” or “late” were used, without defining the criteria) | Yes = 1 / No = 0 |
| Q5 | Are the main findings of the study clearly described? Simple outcome data should be reported for all major findings so the reader can check the major analyses and conclusions. This does not cover statistical tests which are addressed in other questions. | Yes = 1 / No = 0 |
| Q6 | Does the study provide estimates of the random variability in the data for the main outcomes? In non-normal data, interquartile range should be reported. In normal data, standard deviation, standard error or confidence intervals should be reported. | Yes = 1 / No = 0 |
| Q7 | Were the subjects confirmed as non-hormonal contraceptive users, for at least three months prior to participation? | Yes = 1 / No = 0 / Unable to determine = 0 |
| Q8 | Was at least one familiarization trial conducted prior to exercise testing? | Yes = 1 / No = 0 / Unable to determine = 0 |
| Q9 | Were the exercise test conditions adequately standardized (taking into consideration factors including time of day, prior nutritional intake [including caffeine] and prior exercise)? | Yes (all relevant factors) = 2 / Yes (some relevant factors) = 1 / Testing unstandardized = 0 / Unable to determine = 0 |
| Q10 | If any of the results were based on ‘data dredging’ was this made clear? Any analyses that had not been planned at the outset should be clearly no indicated. If retrospective subgroup analyses were reported, answer Yes. | Yes = 1 / No = 0 / Unable to determine = 0 |
| Q11 | Were statistical tests used to assess the main outcomes appropriate? The statistical techniques used must be appropriate to the data and the research question. | Yes = 1 / No = 0 / Unable to determine = 0 |
| Q12 | Were the main outcome measures used accurate (i.e., valid and reproducible)? For studies where the validity and reproducibility of outcome measures are clearly described, the question should be answered Yes. For studies which refer to other work that demonstrates the outcome measures are accurate, answer Yes. | Yes = 1 / No = 0 / Unable to determine = 0 |
| Q13 | Was the order of phase testing randomized? | Yes = 1 / No = 0 / Unable to determine = 0 |
| Q14 | Did the study have sufficient power to detect an a priori specified scientifically important effect at a pre-determined probability threshold? Answer Yes if they included a power calculation, and No if not. | Yes = 1 / No = 0 |
| Q15 | Was study retention > 85%? | Yes = 1 / No = 0 / Unable to determine = 0 |
| Q16 | Was menstrual-cycle phase confirmed using blood-based hormone assessment? | Yes = no downgrade for biochemical phase verification; No = downgrade by one level |
| Q17 | Was ovulation confirmed using prospective ovulation testing? | Yes = no additional downgrade for ovulation verification; No = downgrade by one additional level |

**Note.** Q1–Q15 generate the a priori methodological quality rating. Q16 and Q17 are predefined menstrual-phase verification downgrades.

**Table S1B.** Modified Downs and Black checklist according to McNulty et al. (2020)

| **Item** | **Sawai et al., 2024** | **Funaki et al., 2022** | **Minuzzi et al., 2022** | **Romero-Parra et al., 2020** | **Graja et al. 2022** | **Hackney et al., 2019** | **Williams et al. 2015** | **Chaffin et al., 2011** | **Sipavičienė et al., 2013** | **Parsons et al., 2025** | **Oosthuyse and Bosch, 2017** | **Barba-Moreno et al., 2022** | **Alfaro-Magallanes et al., 2022** | **Zheng et al., 2021** | **Gassner et al., 2026** | **Hicks et al., 2023** | **Silva et al., 2025** | **Yoshida et al., 2026** |
| --- | --- | --- | --- | --- | --- | --- | --- | --- | --- | --- | --- | --- | --- | --- | --- | --- | --- | --- |
| **Q1** | 1 | 1 | 1 | 1 | 1 | 1 | 1 | 1 | 1 | 1 | 1 | 1 | 1 | 1 | 1 | 1 | 1 | 1 |
| **Q2** | 1 | 1 | 1 | 1 | 1 | 0 | 1 | 1 | 0 | 1 | 1 | 1 | 1 | 1 | 1 | 1 | 1 | 1 |
| **Q3** | 1 | 1 | 1 | 1 | 1 | 1 | 1 | 1 | 1 | 1 | 1 | 1 | 1 | 1 | 1 | 1 | 1 | 1 |
| **Q4** | 1 | 1 | 1 | 1 | 1 | 1 | 1 | 1 | 1 | 1 | 1 | 1 | 1 | 1 | 1 | 1 | 1 | 1 |
| **Q5** | 1 | 1 | 1 | 1 | 1 | 1 | 1 | 1 | 1 | 1 | 1 | 1 | 1 | 1 | 1 | 1 | 1 | 1 |
| **Q6** | 1 | 1 | 1 | 1 | 1 | 1 | 1 | 1 | 1 | 1 | 1 | 1 | 1 | 1 | 1 | 1 | 1 | 1 |
| **Q7** | 1 | 1 | 1 | 1 | 1 | 1 | 1 | 1 | 1 | 1 | 1 | 1 | 1 | 1 | 1 | 1 | 1 | 0 |
| **Q8** | 0 | 0 | 0 | 1 | 1 | 0 | 0 | 1 | 1 | 0 | 0 | 0 | 0 | 1 | 1 | 1 | 0 | 0 |
| **Q9** | 2 | 1 | 2 | 0 | 2 | 0 | 1 | 0 | 0 | 1 | 2 | 1 | 2 | 2 | 2 | 1 | 1 | 2 |
| **Q10** | 1 | 1 | 1 | 1 | 1 | 1 | 1 | 1 | 1 | 1 | 1 | 1 | 1 | 1 | 1 | 1 | 1 | 1 |
| **Q11** | 1 | 1 | 1 | 1 | 1 | 1 | 1 | 1 | 1 | 1 | 1 | 1 | 1 | 1 | 1 | 1 | 1 | 1 |
| **Q12** | 1 | 1 | 1 | 1 | 1 | 1 | 1 | 1 | 1 | 1 | 1 | 1 | 1 | 1 | 1 | 1 | 1 | 1 |
| **Q13** | 0 | 1 | 1 | 1 | 1 | 1 | 0 | 0 | 1 | 0 | 1 | 1 | 0 | 1 | 0 | 1 | 1 | 1 |
| **Q14** | 0 | 1 | 0 | 1 | 0 | 0 | 0 | 0 | 0 | 0 | 0 | 0 | 1 | 0 | 1 | 0 | 1 | 0 |
| **Q15** | 0 | 1 | 1 | 1 | 1 | 1 | 1 | 0 | 1 | 0 | 1 | 1 | 1 | 0 | 0 | 1 | 1 | 1 |
| **Q16** | Yes | Yes | No | Yes | Yes | Yes | Yes | Yes | Yes | No | Yes | Yes | Yes | Yes | No | Yes | No | Yes |
| **Q17** | Yes | Yes | No | Yes | Yes | No | No | No | No | Yes | Yes | No | Yes | Yes | No | No | No | No |
| **Rating** | Maintain | Maintain | 2 Down­grades | Maintain | Maintain | 1 Down­grade | 1 Down­grade | 1 Down­grade | 1 Down­grade | 1 Down­grade | Maintain | 1 Down-grade | Maintain | Maintain | 2 Down-grades | 1 Down-grade | 2 Down-grades | 1 Down-grade |
| *A priori* | Moderate | High | High | High | High | Moderate | Moderate | Moderate | Moderate | Moderate | High | High | High | High | High | High | High | High |
| **Final classification** | Moderate | **High** | Low | **High** | **High** | Low | Low | Low | Low | Low | **High** | Moderate | **High** | **High** | Low | Moderate | Low | Moderate |
